# Lactylation stabilizes DCBLD1 activating the pentose phosphate pathway to promote cervical cancer progression

**DOI:** 10.1186/s13046-024-02943-x

**Published:** 2024-01-31

**Authors:** Qingfei Meng, Huihui Sun, Yanghe Zhang, Xiangzhe Yang, Shiming Hao, Bin Liu, Honglan Zhou, Zhi-Xiang Xu, Yishu Wang

**Affiliations:** 1 Key Laboratory of Pathobiology, Ministry of Education, Jilin University, Changchun, 130021 China; 2 Department of Urology, The First Hospital of Jilin University, Changchun, 130021 China; 3 School of Life Sciences, Henan University, Kaifeng, 475000 China

**Keywords:** Lactylation, Pentose phosphate pathway, Autophagy, Cervical cancer, G6PD, DCBLD1, HIF-1α

## Abstract

**Background:**

Discoidin, CUB, and LCCL domain-containing type I (DCBLD1) is identified as an oncogene involved in multiple regulation of tumor progression, but specific mechanisms remain unclear in cervical cancer. Lactate-mediated lactylation modulates protein function. Whether DCBLD1 can be modified by lactylation and the function of DCBLD1 lactylation are unknown. Therefore, this study aims to investigate the lactylation of DCBLD1 and identify its specific lactylation sites. Herein, we elucidated the mechanism by which lactylation modification stabilizes the DCBLD1 protein. Furthermore, we investigated DCBLD1 overexpression activating pentose phosphate pathway (PPP) to promote the progression of cervical cancer.

**Methods:**

DCBLD1 expression was examined in human cervical cancer cells and adjacent non-tumorous tissues using quantitative reverse transcription-polymerase chain reaction, western blotting, and immunohistochemistry. In vitro and in vivo studies were conducted to investigate the impact of DCBLD1 on the progression of cervical cancer. Untargeted liquid chromatography-tandem mass spectrometry (LC–MS/MS) metabolomics studies were used to characterize DCBLD1-induced metabolite alterations. Western blot, immunofuorescence and transmission electron microscopy were performed to detect DCBLD1 degradation of G6PD by activating autophagy. Chromatin immunoprecipitation, dual luciferase reporter assay for detecting the mechanism by which lactate increases DCBLD1 transcription. LC–MS/MS was employed to verify specific modification sites within the DCBLD1 protein.

**Results:**

We found that lactate increased DCBLD1 expression, activating the PPP to facilitate the proliferation and metastasis of cervical cancer cells. DCBLD1 primarily stimulated PPP by upregulating glucose-6-phosphate dehydrogenase (G6PD) expression and enzyme activity. The mechanism involved the increased enrichment of HIF-1α in the DCBLD1 promoter region, enhancing the DCBLD1 mRNA expression. Additionally, lactate-induced DCBLD1 lactylation stabilized DCBLD1 expression. We identified DCBLD1 as a lactylation substrate, with a predominant lactylation site at K172. DCBLD1 overexpression inhibited G6PD autophagic degradation, activating PPP to promote cervical cancer progression. In vivo, 6-An mediated inhibition of G6PD enzyme activity, inhibiting tumor proliferation.

**Conclusions:**

Our findings revealed a novel post-translational modification type of DCBDL1, emphasizing the significance of lactylation-driven DCBDL1-mediated PPP in promoting the progression of cervical cancer.

**Graphical Abstract:**

Schematic illustration of DCBLD1-induced G6PD-mediated reprogramming of PPP metabolism in promoting cervical cancer progression.

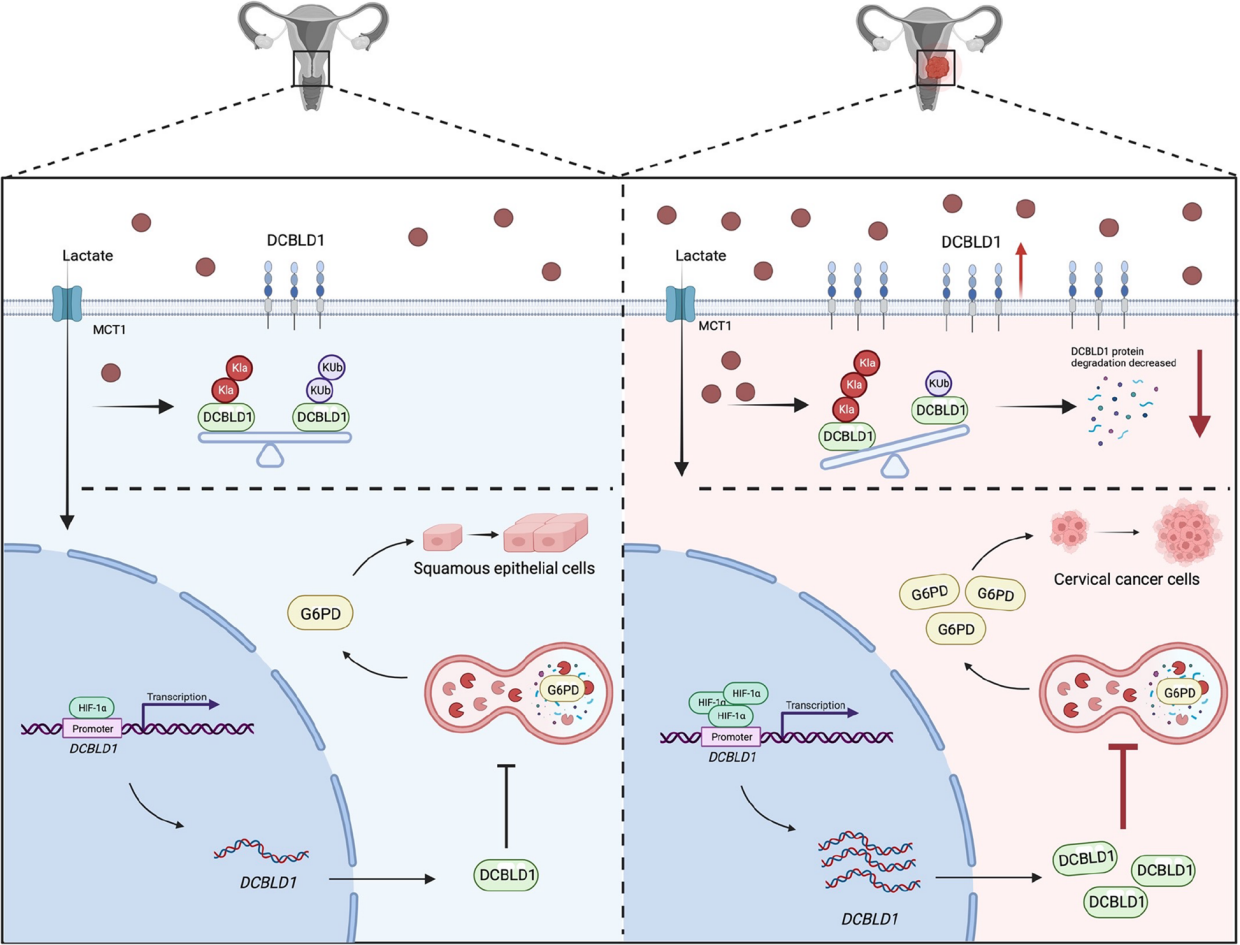

**Supplementary Information:**

The online version contains supplementary material available at 10.1186/s13046-024-02943-x.

## Introduction

Cervical cancer ranks as the fourth most prevalent cancer among women, underscoring the significance of exploring the pathogenesis and targeted therapy. Discoidin, CUB, and LCCL Domain-containing type I (DCBLD1) is widespread and sequence-conserved in mammals, with a total length of 715 amino acids, an intracellular portion with a conserved signaling sequence [1–3]. Although the intracellular region has no specific modular structural domain, the intracellular scaffolding domains of DCBLD1 harbor eight intracellular binding motifs of the SH2 domains of the signaling adaptors CT10 regulator of kinase (CRK) and CRK-like (CRKL) [4] as well as several phosphorylation, acetylation and ubiquitination sites [5, 6]. DCBLD1 is involved in receptor tyrosine kinase signaling [4]. Nonreceptor tyrosine kinases FYN and ABL can lead to the phosphorylation of the YXXP motif of DCBLD1, which results in inducing the interaction between DCBLD1 and the SH2 domain of CRK/CRKL [3, 6]. Due to their lack of enzymatic activity, DCBLD1 protein is thought to function as scaffolds for signaling centers at the plasma membrane. Overexpression of DCBLD1 is associated with poor prognosis in head and neck squamous cell carcinoma, non-small cell lung cancer, breast cancer, pancreatic cancer, and renal cell carcinoma [7–10], its specific role in the development of cervical cancer remains unclear.

Abnormal metabolism is one of the hallmarks of tumors [11]. They tend to favor energy production via glycolysis over oxidative phosphorylation, even in the presence of oxygen [12]. Lactate, a glycolytic metabolite, serves as a signaling molecule or metabolic substrate and participates in various crucial processes, including glucose metabolism, fatty acid synthesis, redox homeostasis, and post-translational modifications [13]. Lysine lactylation (Kla) is novel lactate-driven post-translational modifications [14]. Studies on lactylation have primarily focused on histone and non-histone proteins. In particular, histone lactylation plays a significant role in regulating macrophage polarization and the reprogramming of embryonic fibroblasts into pluripotent stem cells [15]. Non-histone lactylation modifications have been increasingly recognized for their role in tumor progression. For example, the lactylation of adenylate kinase 2 has been shown to enhance the proliferation and metastasis of hepatocellular carcinoma (HCC) [16]. Lactylation is also involved in the regulation of CEA Adhesion Molecule-6 protein stability in colorectal cancer [17]. Despite these advancements, targets of lactylation in cervical cancer remain elusive. Further investigation is required to elucidate the functional implications of lactylation in the pathogenesis of cervical cancer.

The pentose phosphate pathway (PPP) branches off from glycolysis and comprises the oxidative PPP (oxiPPP) and non-oxidized PPP (non-oxiPPP) [18]. Within this pathway, G6PD, functioning as the sole rate-limiting enzyme in PPP, mediates the oxiPPP, primarily producing NADPH to maintain intracellular redox homeostasis. The non-oxidative PPP mainly serve as substrates for synthesizing nucleotides. Compelling evidence suggests that PPP exhibits aberrant activation in tumor cells, supplying them with NADPH and essential metabolites [19]. We previously found that glycolysis and PPP are overactivated in cervical cancer [20–22]. However, whether DCBLD1 triggers PPP activation to promote the proliferation of cervical cancer cells remains unclear.

Autophagy is a conserved catabolic mechanism responsible for degrading cytoplasmic components, abnormal proteins, and impaired organelles [23]. This process is characterized by the development of double-membrane structures called "autophagosomes," which subsequently fuse with vesicles or lysosomes to facilitate the degradation of their contents [24]. Autophagy is hyperactivated in cervical cancer progression [25]. Autophagy regulates glycolysis via selective degradation of HK2 [26]. Additionally, G6PD is degraded by chaperone-mediated autophagy processes [27]. However, whether DCBLD1 activates autophagy to degrade G6PD, leading to metabolic reprogramming needs to be further investigated.

## Results

### DCBLD1 promotes migration, invasion, and growth of cervical cancer cells

**Fig. 1:**
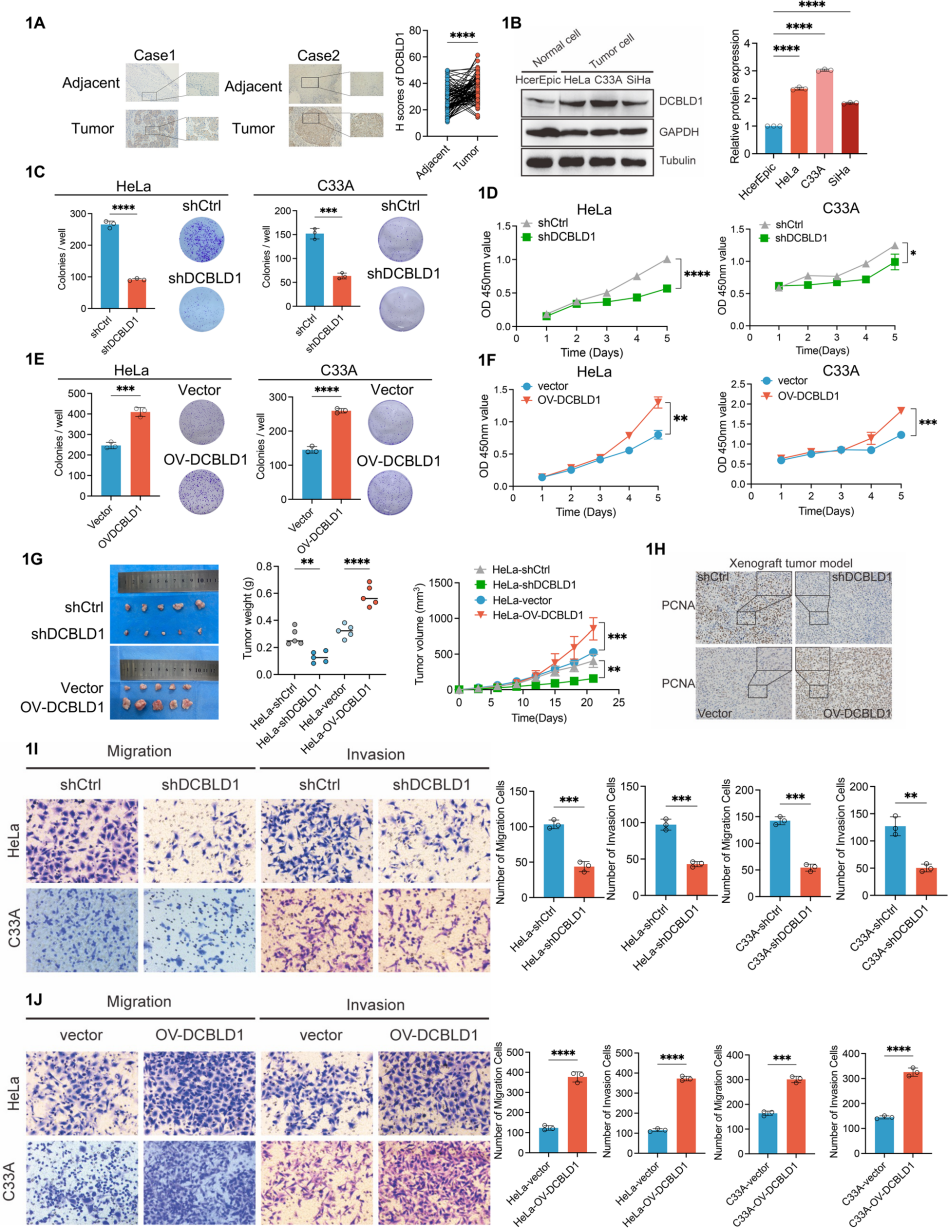
DCBLD1 promotes cervical cancer progression in vivo and in vitro. **A** IHC staining for DCBLD1 was performed on tumor sections of human cervical cancer and adjacent normal tissue. Subsequently, H-scores were plotted. *n* = 50. **B** Immunoblotting was used to assess DCBLD1 levels in lysates extracted from various cell lines, including human normal cervical epithelial (HcerEpic) and cervical cancer (HeLa, C33A, and SiHa) cells. GAPDH and Tubulin are used as loading controls. The graph on the right is a statistical plot of the western blot. **C**, **E** The cell proliferation rate was determined using a clone formation assay. **D**, **F** Cell viability was determined using CCK8 assays. **G** Xenograft tumor images derived from HeLa cells. *n* = 5 mice per group. Tumor growth curves of the different groups. Weight of the excised tumors in each group. **H** Sections of tumor tissue from the xenograft tumor model were subjected to PCNA staining. **I**–**J** Transwell migration and invasion assays were performed on HeLa and C33A cells, stably expressing shCtrl, shDCBLD1, vector, and OV-DCBLD1. Data are presented as mean ± SD. Statistical significance was assessed using an unpaired t test (for panels **A**, **C**–**F**, and **G** for tumor volume, as well as **I**–**J**), a one-way ANOVA with Dunnett's multiple comparisons test (**B**), or a one-way ANOVA with the Brown-Forsythe test (G for tumor weight). (****, *p* < 0.0001; ***, *p* < 0.001; **, *p* < 0.01; *, *p* < 0.05).Scale bar: 50 µm

To investigate whether DCBLD1 is involved in the progression of cervical cancer, we first analyzed the expression of DCBLD1 through The Cancer Genome Atlas (TCGA) database, and the results showed that patients with high DCBLD1 expression have shorter survival (Supplementary Fig. 1A). Consistently, the Gene Expression Omnibus (GEO) (GSE39001 and GSE75132) database was used to further validate DCBLD1 expression, which was highly expressed in cervical cancer compared to paired normal tissues (Supplementary Fig. 1B). We used immunohistochemistry (IHC) to probe the presence of DCBLD1 in a large collection of normal, carcinomas of the cervix. As shown in Fig. 1A, normal cervical tissues bore a low level of DCBLD1, whereas Cancer tissues exhibited significantly higher signals. Normal cervical cell and cervical cancer cells were used to detect the expression of DCBLD1, we found that DCBLD1 mRNA expression was significantly higher in HeLa cells compared to HcerEpic cells (Supplementary Fig. 1C). Consistently, the protein expression of DCBLD1 was significantly higher in cervical cancer cells than in normal cervical epithelial cells (Fig. 1B).

To determine whether DCBLD1 had a direct influence on cervical cancer cell phenotypes, we firstly evaluated the proliferation of HeLa and C33A cells following DCBLD1 knockdown, achieved through the use of short hairpin RNA (shRNA) (Supplementary Fig. 2A, B). The results showed that HeLa and C33A cells with DCBLD1 knockdown (KD) formed smaller colonies and exhibited diminished cell viability compared to the parental cells (Fig. 1C, D). Conversely, DCBLD1 overexpression in HeLa and C33A cells substantially enhanced cell viability and colony formation (Fig. 1E, F). To validate these results in vivo, we conducted experiments in the xenograft models. Nude mice were administered injections of control, ov-DCBLD1, or DCBLD1-KD HeLa cells. Our findings revealed that tumors originating from DCBLD1-KD cells were significantly smaller than those derived from control cells. In contrast, DCBLD1 overexpression substantially amplified tumor volume (Fig. 1G). Additionally, we examined the expression of proliferating cell nuclear antigen (PCNA) in DCBLD1-OV and DCBLD1-KD tumor tissues generated from HeLa cells using IHC. The findings revealed that PCNA signals in DCBLD1-KD tumors were weaker than in those in control, while stronger PCNA expression was observed in DCBLD1-OV tumors than in control (Fig. 1H). Subsequently, we assessed the migration and invasion capabilities of HeLa/C33A-KD and HeLa/C33A-OV cells. The depletion of DCBLD1 markedly reduced cell migration than in the control cells, as demonstrated in the transwell assay. Similarly, the ability of HeLa/C33A cells to transverse the Matrigel membrane was substantially reduced upon DCBLD1 KD (Fig. 1L and J). In a parallel wound-healing (scratch) assay, we observed that DCBLD1-depleted HeLa/C33A cells exhibited slower migration in closing the wound than DCBLD1-intact cells (Supplementary Fig. 2C). Collectively, these findings suggest that DCBLD1 promotes cervical cancer cell proliferation, migration, and invasion in vivo and in vitro.

### DCBLD1 activates the PPP

**Fig. 2:**
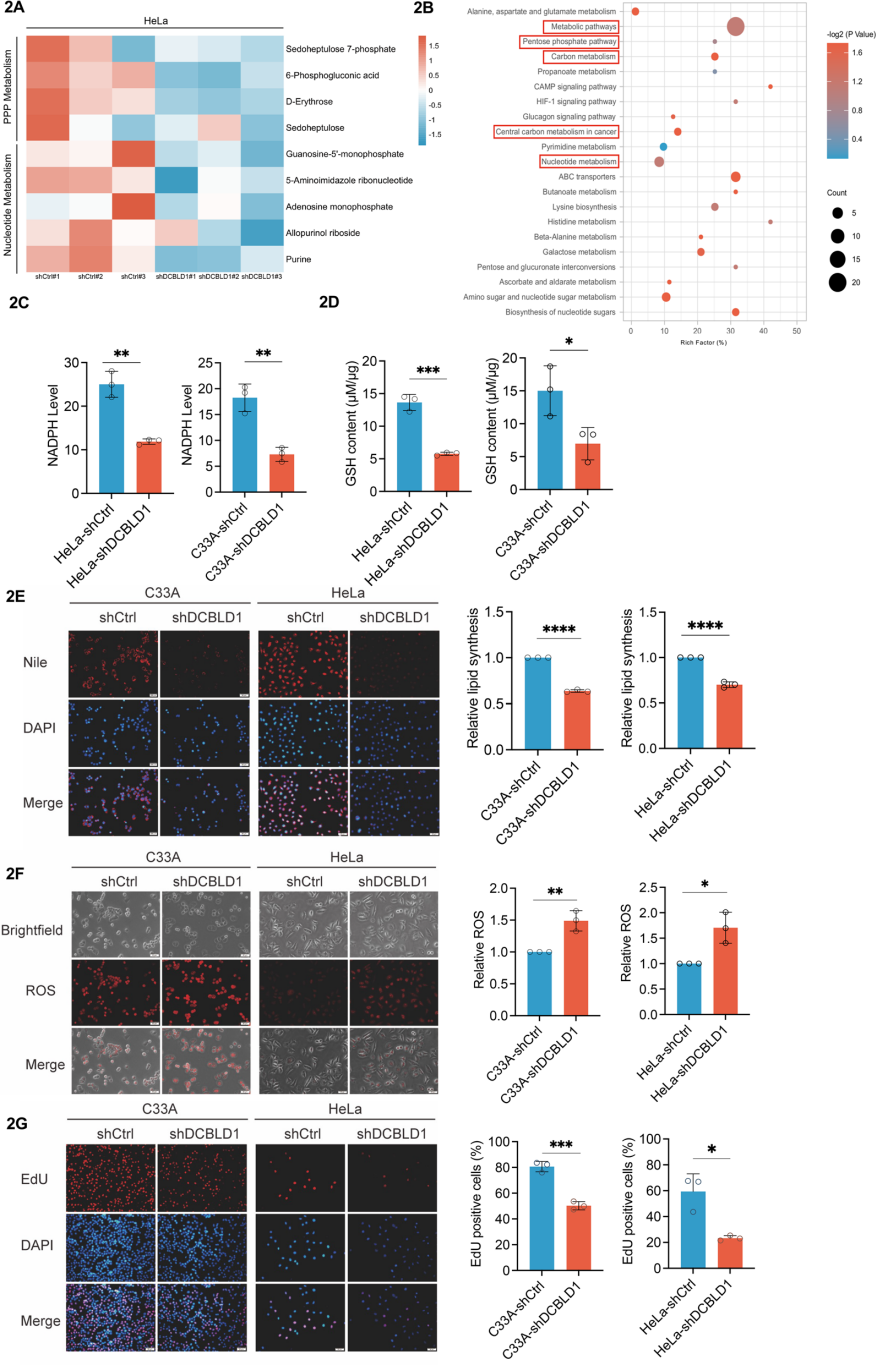
DCBLD1 knockdown inhibits PPP. **A** A heatmap illustrating the down-regulated metabolites in HeLa cells expressing shDCBLD1 compared to shCtrl cells. **B** KEGG enrichment analysis of differential metabolites. **C**–**D** Intracellular NADPH and GSH levels were assayed in HeLa and C33A cells expressing shCtrl or shDCBLD1. **E** Detection of lipid synthesis by Nile Red staining. Representative images (left) and average fluorescence quantification results (right) are shown. **F** Reactive oxygen species production levels were detected using ROS staining. Representative images (left) and the average fluorescence quantification results (right) are shown. **G** DNA synthesis in HeLa and C33A cells expressing shCtrl or shDCBLD1 was determined using EdU. EdU-positive cell proportion was analyzed using ImageJ. Representative images (left) and the quantification results (right) are shown. Data are presented as mean ± SD. Statistical significance was assessed using an unpaired t test (**C**-**G**). (****, *p* < 0.0001; ***, *p* < 0.001; ***, *p* < 0.001; **, *p* < 0.01; *, *p* < 0.05). Scale bar: 50 µm

To identify the metabolic pathways affected by DCBCLD1 in facilitating cell proliferation, we utilized DCBLD1 KD or control HeLa cells to assess differences in metabolite profiles using liquid chromatography-mass spectrometry (LC–MS). The analysis revealed a decrease in the levels of substrates associated with nucleotide and PPP metabolisms (Fig. 2A). Furthermore, KEGG analysis revealed enriched metabolites involved in metabolic pathways, including PPP, carbon metabolism, central carbon metabolism in cancer, and nucleotide metabolism (Fig. 2B). Subsequently, we investigated whether DCBLD1 activates the PPP. We found a significant decrease in the expression of the PPP metabolites NADPH and GSH in cells subjected to DCBLD1 KD than in control cells (Fig. 2C, D). NADPH is implicated in lipid synthesis and the maintenance of intracellular redox homeostasis. Nile Red staining confirmed that the inhibition of lipid synthesis occurred due to DCBLD1 KD (Fig. 2E). DCBLD1 KD in C33A and HeLa cells resulted in the accumulation of reactive oxygen species (ROS) levels compared to those in the control cell (Fig. 2F). PPP metabolites can furnish substrates for DNA synthesis. Consistent with this, DCBLD1 KD significantly inhibited DNA synthesis (Fig. 2G).

**Fig. 3:**
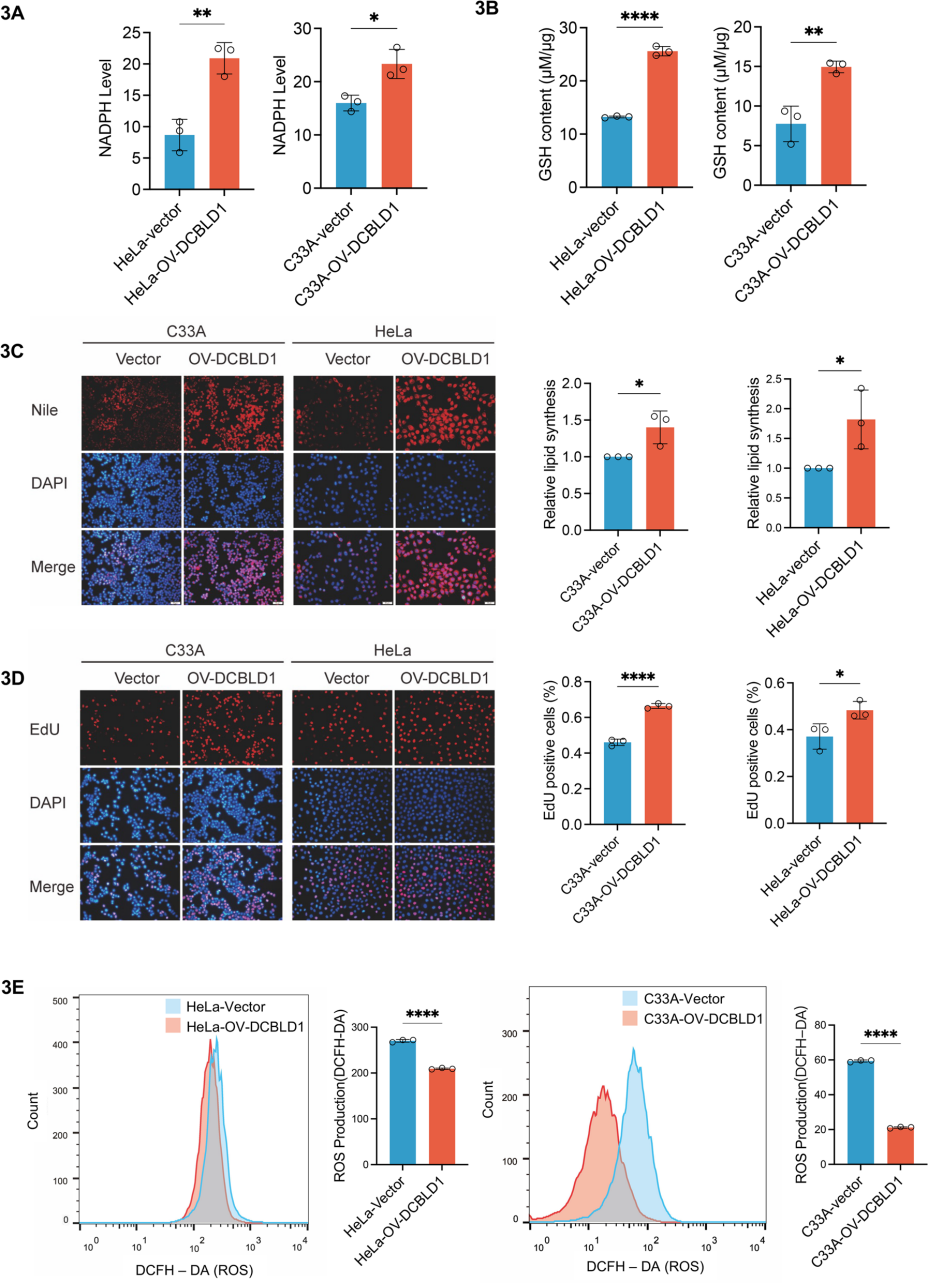
DCBLD1 overexpression activates the PPP. **A**, **B** Intracellular NADPH and GSH levels were measured in HeLa and C33A cells expressing either the vector or OV-DCBLD1. **C** Lipid synthesis detection using Nile Red staining. Representative images (left) and the average fluorescence quantification results (right) are shown. **D** DNA synthesis in HeLa and C33A cells expressing the vector or OV-DCBLD1 was determined using EdU. EdU-positive cell proportion was analyzed using ImageJ. Representative images (left) and the quantification results (right) are shown. **E** DCFH-DA was used to examine ROS in the cytoplasm of HeLa and C33A cells. Representative images obtained through flow cytometry are shown (left), with corresponding histograms presented on the right side. Data are shown as mean ± SD. Statistical significance was assessed using an unpaired t test (**A**-**E**). (****, *p* < 0.0001; **, *p* < 0.01; *, *p* < 0.05). Scale bar: 50 µm

To validate the activation of the PPP by DCBLD1, we engineered cells to overexpress DCBLD1. The results showed a significant elevation in NADPH and GSH levels in HeLa and C33A cells overexpressing DCBLD1 (Fig. 3A, B). Additionally, DCBLD1 promoted lipid (Fig. 3C) and DNA syntheses (Fig. 3D) while reducing ROS (Fig. 3E) in HeLa and C33A cells. These findings collectively suggest that DCBLD1 activates PPP, increases NADPH production, maintains intracellular redox homeostasis, and enhances DNA synthesis.

### DCBLD1 activates PPP mediated by G6PD

**Fig. 4:**
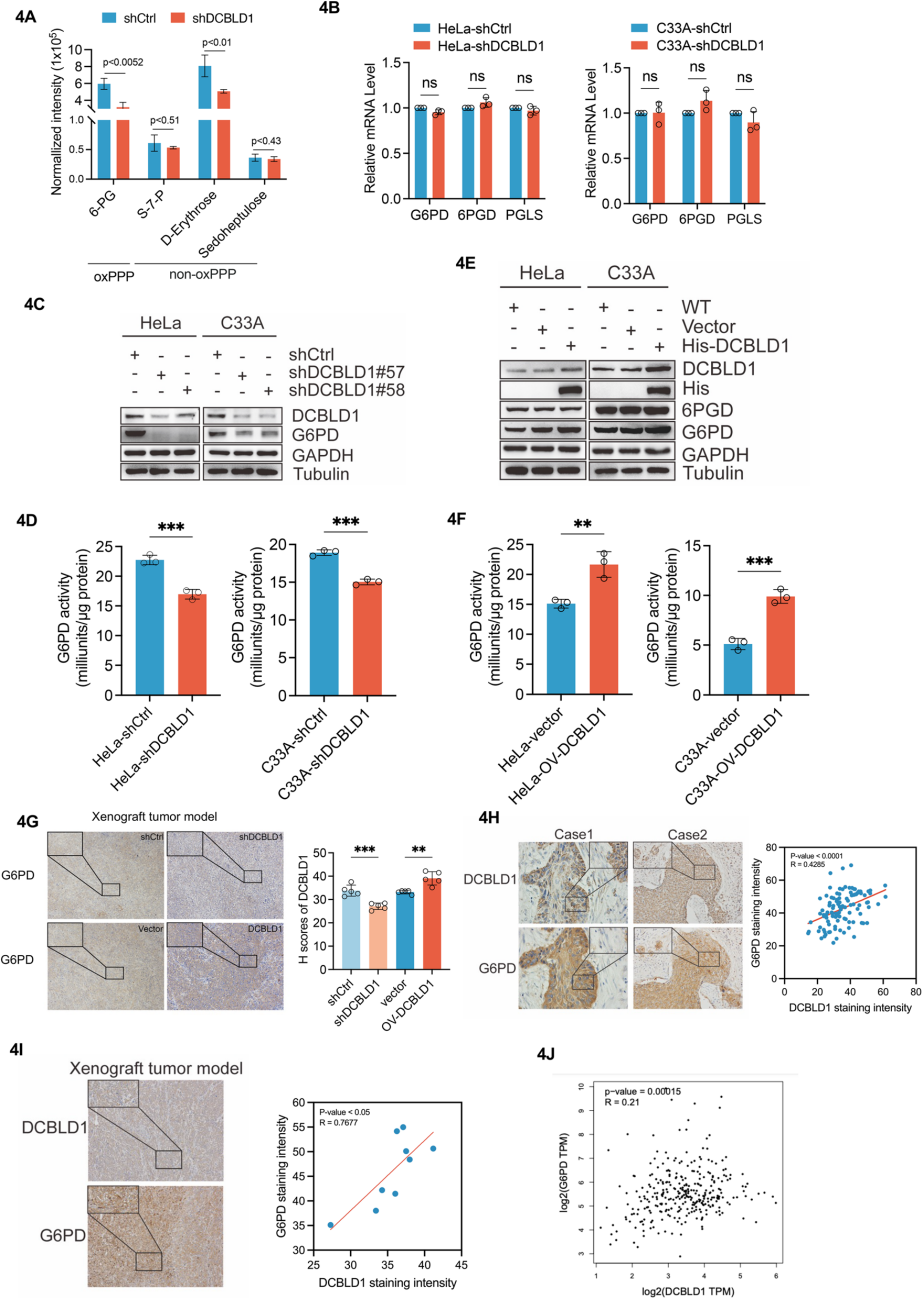
G6PD mediated PPP activation by DCBLD1. **A** A histogram depicting the downregulated levels of pentose phosphate pathway metabolites in HeLa cells expressing shDCBLD1 compared to shCtrl cells. **B** Determination of the mRNA levels of pentose phosphate pathway-related enzymes in transduced cells by qPCR. **C**, **E** Immunoblotting was used to detect DCBLD1, His, 6PGD, and G6PD levels in cell lysates. GAPDH was used as a loading control. **D**, **F** G6PD activity was examined in these cells. **G** IHC staining for DCBLD1 and G6PD was performed on tumor sections obtained from a xenograft tumor model. **H** IHC staining for DCBLD1 and G6PD was perform on tumor sections of human cervical cancer and corresponding adjacent normal tissue. **I** IHC staining for DCBLD1 and G6PD was performed on tumor tissue sections from the xenograft tumor model at the same site. The area of optical density/positive staining area was quantified using ImageJ and further analyzed using GraphPad Prism. **J** DCBLD1 and G6PD correlation analysis using TCGA database. Data are presented as mean ± SD. Statistical significance was assessed using the multiple unpaired t test (**B**), the unpaired t test (**D** and **F**), a one-way ANOVA with Tukey's multiple comparisons test (**G**), and correlation analysis (H and I). (***, *p* < 0.001; **, *p* < 0.01; NS, no significance). Scale bar: 50 µm

To investigate the targets of PPP activation by DCBLD1, we analyzed PPP metabolites using LC–MS. We observed a substantial reduction in oxiPPP metabolites expression in DCBLD1 KD cells (*p* < 0.0052), whereas the non-oxPPP metabolites remained unchanged, except for D-Erythrose (Fig. 4A). We then examined enzymes associated with oxiPPP, including G6PD, 6-phosphogluconate dehydrogenase (6PGD), and 6-phosphogluconolactonase, Analysis of mRNA expression for oxiPPP enzyme showed minimal alteration in C33A and HeLa cells upon DCBLD1 KD (Fig. 4B). However, G6PD protein expression and enzymatic activity were reduced (Fig. 4C, D). In contrast, the overexpression of DCBLD1 led to a significant increase in G6PD expression and enzymatic activity (Fig. 4E, F). To validate these findings in vivo, a xenograft experiment was conducted involving nude mice. These mice were administered control HeLa cells or cells expressing DCBLD1 or with DCBLD1 knockdown. Subsequently, xenograft tumors were used for IHC analysis to detect DCBLD1 and G6PD expressions. As consistently observed, the KD of DCBLD1 in the tissues resulted in reduced G6PD expression, while tissues overexpressing DCBLD1 exhibited robust G6PD signals (Fig. 4G). In human cervical cancer tissues and xenograft tumor samples, DCBLD1 positively correlated with G6PD (Fig. 4H, I). Furthermore, DCBLD1 expression was positively associated with G6PD expression based on information from TCGA database (based on the pairwise correlation analysis results from the gene expression profiling interactive analysis: http://gepia.cancer-pku.cn/) (Fig. 4J). These findings indicate that DCBLD1 activates G6PD.

### DCBLD1 activates autophagy-degrading G6PD

**Fig. 5:**
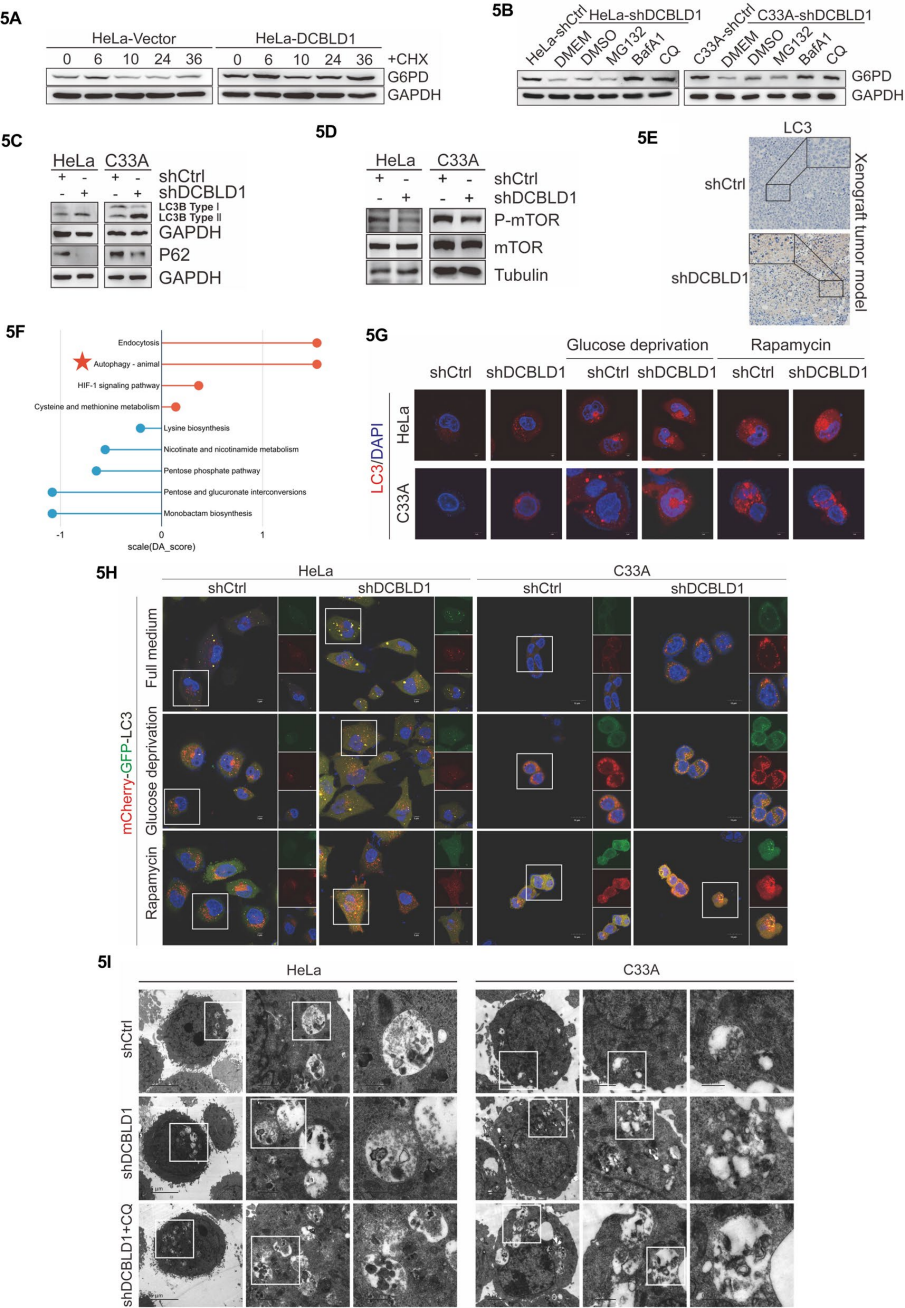
DCBLD1 inhibits the autophagic degradation of G6PD. **A** HeLa cells expressing both the vector and DCBLD1 were treated with 10 μg/ml CHX for the specified durations. Whole-cell extracts (WCE) were collected for immunoblotting to detect G6PD levels in the cells. GAPDH was used as a loading control. **B** HeLa and C33A cells expressing shDCBLD1 were treated with MG132 (10 μm), BafA1 (200 nM), and CQ (25 μm) for 24 h. WCE were collected for immunoblotting to detect G6PD in the cells. GAPDH was used as a loading control. **C**, **D** HeLa and C33A cells expressing shCtrl and shDCBLD1, and WCE were collected for immunoblotting to detect LC3 I, LC3 II, P62, and mTOR/P-mTOR levels in the cells. GAPDH and Tubulin were used as loading controls. **E** Tumor sections from the xenograft tumor model were stained with IHC for LC3. **F** A statistical plot illustrating metabolite changes in HeLa cells expressing shDCBLD1 than those expressing shCtrl. **G** HeLa and C33A cells expressing shCtrl and shDCBLD1 were cultured under conditions of glucose deprivation and treated with Rapamycin (25 nM) for 24 h. LC3 puncta formation was subsequently detected through immunostaining. Scale bar: 1 μm. **H** Representative confocal images of GFP-LC3 and mRFP-LC3 distribution in HeLa and C33A cells transfected with DCBLD1 shRNA vector, both with or without rapamycin treatment and glucose deprivation for 24 h. Scale bar: 5 μm. **I** Representative images illustrating the autophagic structures in HeLa and C33A cells transfected with the DCBLD1 shRNA vector, both with or without CQ treatment for 24 h (25 μM). Scale bar: 2 μm, 1 μm, and 0.5 μm

The KD of DCBLD1 resulted in decreased G6PD protein levels in C33A and HeLa cells without affecting G6PD mRNA levels (Fig. 4B, C). Therefore, we hypothesize that the regulation of G6PD expression is more likely to occur at the post-transcriptional level rather than transcriptionally. To investigate this further, HeLa cells were treated with the protein synthesis inhibitor cycloheximide (CHX). The results showed that DCBLD1 overexpression markedly extended the half-life of G6PD (Fig. 5A). Eukaryotic cells employ three primary mechanisms to regulate protein degradation: the proteasome, lysosome, and autolysosome pathways [28]. We then proceeded to identify the specific system responsible for G6PD degradation. The findings revealed that the decrease of G6PD protein levels due to DCBLD1 deficiency was reversed by treatment with autophagy inhibitors, chloroquine (CQ) and bafilomycin A1 (BafA1), but not with the proteasome inhibitor MG132 (Fig. 5B). To assess the inhibitory effect of DCBLD1 on autophagy within these two cell lines, changes in P-mTOR, MAP1LC3B/LC3B, and SQSTM1/P62 proteins were observed. All three serve as pivotal autophagy indicators. We observed that knockdown of DCBLD1 led to an increased in LC3B-II and a reduction in p62 and P-mTOR (Fig. 5C, D). Consistent with these findings, in xenograft tumors derived from harboring Hela-shCtrl and Hela-shDCBLD1 cells, we observed elevated LC3B expression in tissues with DCBLD1 KD than in control (Fig. 5E). Further KEGG analysis of differential metabolites (shDCBLD1 vs shCtrl) revealed significant upregulation in the autophagy pathway (Fig. 5F). These outcomes observed in C33A and HeLa cells suggest that DCBLD1 KD activates autophagy.

In line with the alterations in LC3B-II protein levels, the formation of red fluorescent protein-light chain 3 (RFP-LC3) puncta structures, indicating the accumulation of autophagosomes, were increased during glucose deprivation in RFP-LC3 transiently expressing C33A and HeLa cells (middle panels in Fig. 5G). Furthermore, the abundance of LC3 puncta structures increased following rapamycin treatment (right panels in Fig. 5G).

To monitor the autophagic flux regulated by DCBLD1 KD dynamically, a tandem GFP-RFP-LC3 plasmid was introduced into C33A and HeLa cells. During glucose deprivation, we observed a greater abundance of red puncta (RFP-LC3) than green puncta (GFP-LC3) in GFP-RFP-LC3 transiently expressed C33A and HeLa cells (Fig. 5H), suggesting the activation of autophagic flux during starvation (acidic lysosomes quenched GFP-LC3 puncta due to successful fusion between autophagosomes and lysosomes). Transmission electron microscopy further revealed that the KD of DCBLD1 augmented autophagic structure formation. Moreover, the combined application of autophagy inhibitor CQ amplified the abundance of these autophagic structures (Fig. 5I). Collectively, these findings indicate that DCBLD1 KD activates autophagy and facilitates the degradation of G6PD via the autophagic lysosomal pathway.

### G6PD is critical for DCBLD1-mediated tumor proliferation in vivo and vitro

**Fig. 6:**
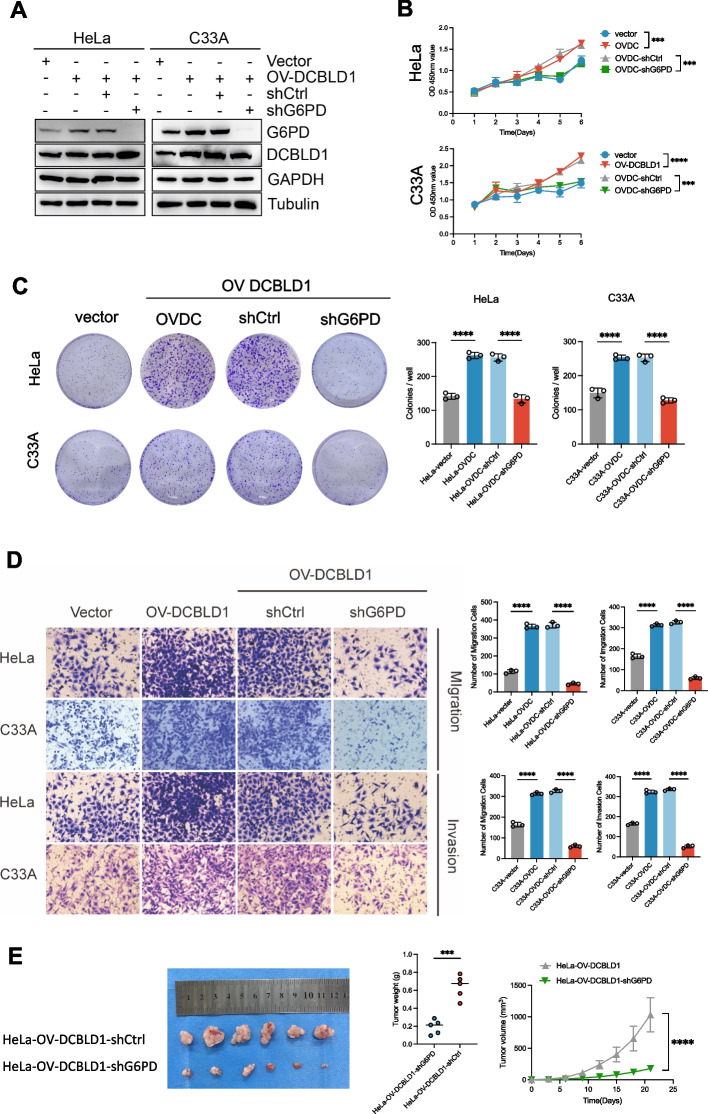
G6PD knockdown blocks DCBLD1-induced increases in cell proliferation, migration, and invasion. **A** Immunoblotting was used to detect DCBLD1 and G6PD levels in cell lysates. GAPDH was used as a loading control. **B** Cell viability was determined using CCK8 assays. **C** Cell proliferation rate was determined using clone formation assay. **D** Transwell migration and invasion assays were performed on HeLa and C33A cells, stably expressing vector, OV-DCBLD1, OV-DCBLD1-shCtrl, and OV-DCBLD1-shG6PD. **E** Xenograft tumor images derived from HeLa cells. *n* = 6 mice per group. Tumor growth curves of the different groups. Weight of the excised tumors in each group. Data are presented as mean ± SD. Statistical significance was assessed using a one-way ANOVA with the Brown-Forsythe test (**B**, **C**, and **D**) or an unpaired t test (**E**). (****, *p* < 0.0001; ***, *p* < 0.001). Scale bar: 50 µm

To determine the significance of the G6PD-mediated PPP pathway for DCBLD1-regulated cell proliferation, migration, and invasion, G6PD was knocked down in C33A and HeLa cells harboring the overexpression of DCBLD1. This was achieved by infecting lentiviruses expressing shG6PD (Fig. 6A). The results showed that G6PD KD reversed the DCBLD1-induced enhancement in cell proliferation, migration, and invasion (Fig. 6B-D). Further, in vivo validation experiments revealed that the tumors originating from cells with G6PD KD were significantly smaller than those originating from control cells (Fig. 6E).

**Fig. 7:**
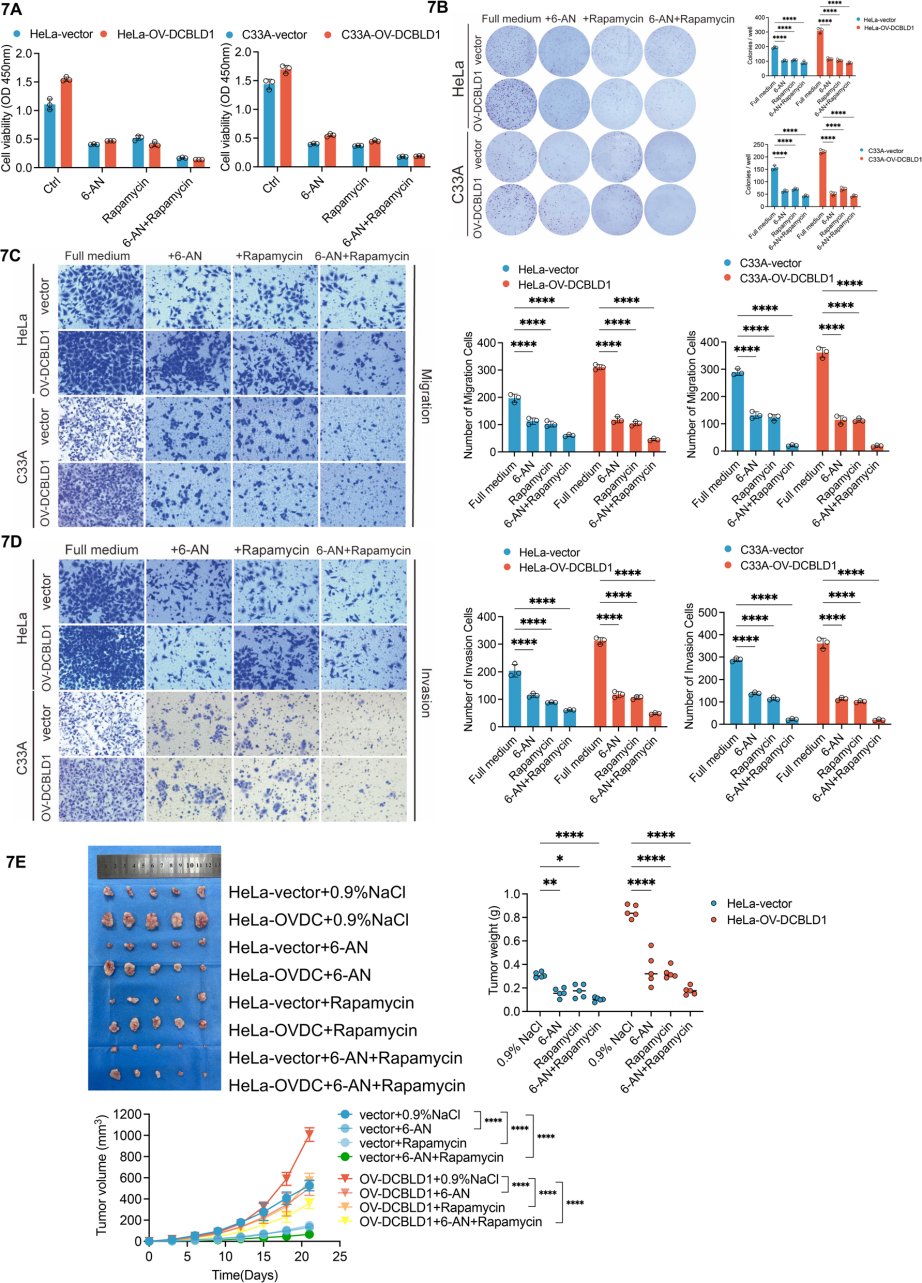
G6PD pharmacological inhibition blocks DCBLD1-induced increases in cell proliferation, migration, and invasion. **A** HeLa and C33A cells, which were stably expressing both the vector and OV-DCBLD1, were treated with 6-AN (120 nM) and rapamycin (25 nM) either individually or in combination. **B** Cell viability was determined through CCK8 assays, while the cell proliferation rate was determined using the clone formation assay. **C**, **D** Transwell migration and invasion assays were performed on HeLa and C33A cells, stably expressing the vector and OV-DCBLD1 after 24 h treatment with 6-AN (120 nM) and Rapamycin (25 nM) individually or in combination. **E** A total of 1 × 10^6^ HeLa-vector and HeLa-OV-DCBLD1 cells were injected subcutaneously into the right abdomen of 4- to 5-week-old female nude mice (*n* = 5). Images show xenograft tumors in nude mice after treatment with 4 mg/kg/2d 6-An and 2 mg/kg/2d Rapamycin individually or in combination. Tumor size was measured every 5 days for 3 weeks. Scale bars: 10 mm. *n* = 6 mice per group. Tumor growth curves of the different groups. Weight of the excised tumors in each group. Data are presented as mean ± SD. Statistical significance was assessed using a one-way ANOVA with the Brown-Forsythe test (**A**), a two-way ANOVA with Šídák's multiple comparisons test (**B**, **C**, and **D**; **E** for tumor weight), and a one-way ANOVA with Tukey's multiple comparisons test (E for tumor volume). (****, *p* < 0.0001; **, *p* < 0.01; *, *p* < 0.05). Scale bar: 50 µm

We subsequently investigated the role of pharmacological inhibition of G6PD, in combination with autophagy inhibitors, on inhibiting DCBDL1-mediated tumor proliferation. Treatment with 6-An (G6PD inhibitors) or rapamycin was found to inhibit cell viability, migration, and invasiveness. Furthermore, the combined treatment approach exhibited a greater reduction in cell viability, migration, and invasiveness in C33A and HeLa cells (Fig. 7A–D). To substantiate these observations in vivo, a xenograft experiment was conducted using nude mice. The mice were injected with control HeLa cells or cells expressing DCBLD1. 6-An and/or Rapamycin was administered intraperitoneally after ensuring successful tumor establishment. The results showed that tumor sizes became smaller following treatment with 6-An or rapamycin than in control. Moreover, the combined treatment yielded an even smaller tumor volume (Fig. 7E, F).

### L-lactate transcriptionally regulates DCBLD1 expression by activation of HIF-1α

**Fig. 8:**
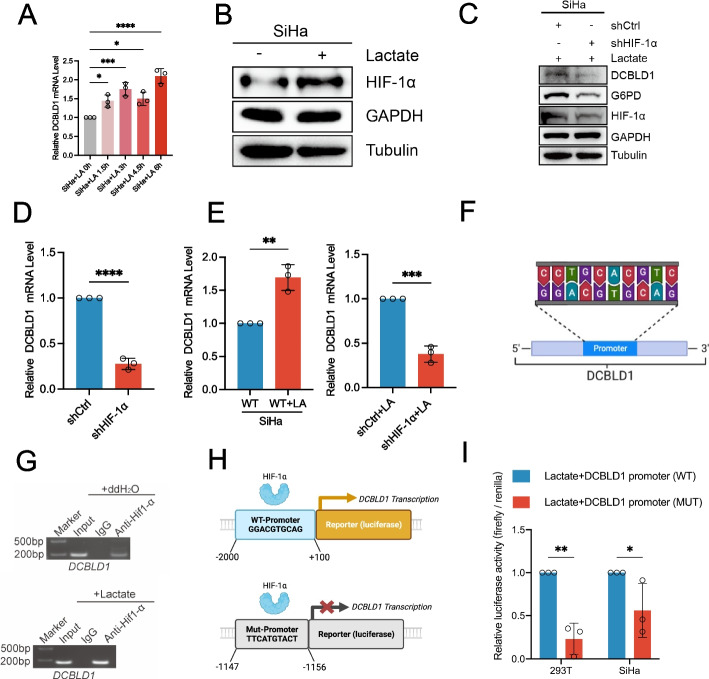
L-lactate-activated Hif-1α promotes DCBLD1 transcription. **A** DCBLD1 mRNA levels in SiHa cells, treated with the addition of sodium L-lactate (25 mM) for varying durations, were determined using qPCR. **B**, **C** Immunoblotting was performed to determine the Hif-1α level after a 6 h treatment with sodium L-lactate (25 mM). GAPDH was used as a loading control. **C** Immunoblotting was performed to determine Hif-1α, G6PD, and DCBLD1 levels in SiHa cells transfected with DCBLD1 shRNA vector, both with or without treatment with sodium L-lactate (25 mM). **D**, **E** DCBLD1 mRNA levels were determined using qPCR. **F** JASPAR was used to predict Hif-1α binding sites to the DCBLD1 promoter. **G** Chip assay for Hif1α binding to the DCBLD1 promoter, with/without sodium L-lactate (25 mM) treatment. **H** Construction of plasmids for wild-type and mutant (at the 1147–1156) luciferase reporter genes region of the DCBLD1 promoter. **I** A luciferase reporter gene assay was performed to validate the Hif1α binding site on the DCBLD1 promoter. Data are presented as the mean ± SD. Statistical significance was assessed using a one-way ANOVA with the Brown-Forsythe test (**A**), an unpaired t test (**D** and **E**), or a two-way ANOVA with Šídák's multiple comparisons test (**I**). (****, *p* < 0.0001; ***, *p* < 0.001; **, *p* < 0.01; *, *p* < 0.05)

Subsequently, we explored the mechanisms underlying the elevated expression of DCBLD1 in cervical cancer. We found that L-lactate, one of the most abundant metabolites in the tumor microenvironment generated through glycolysis, induced time-dependent upregulation in the mRNA expression of DCBLD1 (Fig. 8A). We predicted HIF-1α as a potential transcription factor regulating DCBLD1 through the database (http://genome.ucsc.edu/). Treatment with L-lactate was observed to elevate HIF-1α expression (Fig. 8B). Furthermore, HIF-1α-KD and concurrent application of L-lactate nullified the increase in DCBLD1 transcription and protein expression (Fig. 8C–E). These findings suggest that HIF-1α mediates L-lactate regulation of DCBLD1 transcription. We also predicted HIF-1α binding to the DCBLD1 promoter region through the JASPER website (https://jaspar.genereg.net/) (Fig. 8F). Subsequently, chromatin immunoprecipitation followed by PCR using anti- HIF-1α antibodies was performed to assess the regulatory function of HIF-1α on DCBLD1 expression. The findings revealed an enrichment of HIF-1α in the promoter regions of DCBLD1. Furthermore, the presence of L-lactate was observed to further enhance HIF-1α enrichment in the DCBLD1 promoter region (Fig. 8G). Subsequently, wild-type and mutant luciferase reporter gene plasmids were constructed (Fig. 8H). The luciferase reporter gene assay revealed that the co-administration of L-lactate effectively activated DCBLD1 promoter activity in SiHa cells expressing the wild-type plasmid but not in those expressing the mutant plasmid (Fig. 8I). Collectively, these findings indicate that L-lactate functions as a signaling molecule, activating HIF-1α to enhance DCBLD1 expression.

### L-Lactate inhibits DCBLD1 degradation by increasing directly DCBLD1 lactylation modifications

**Fig. 9:**
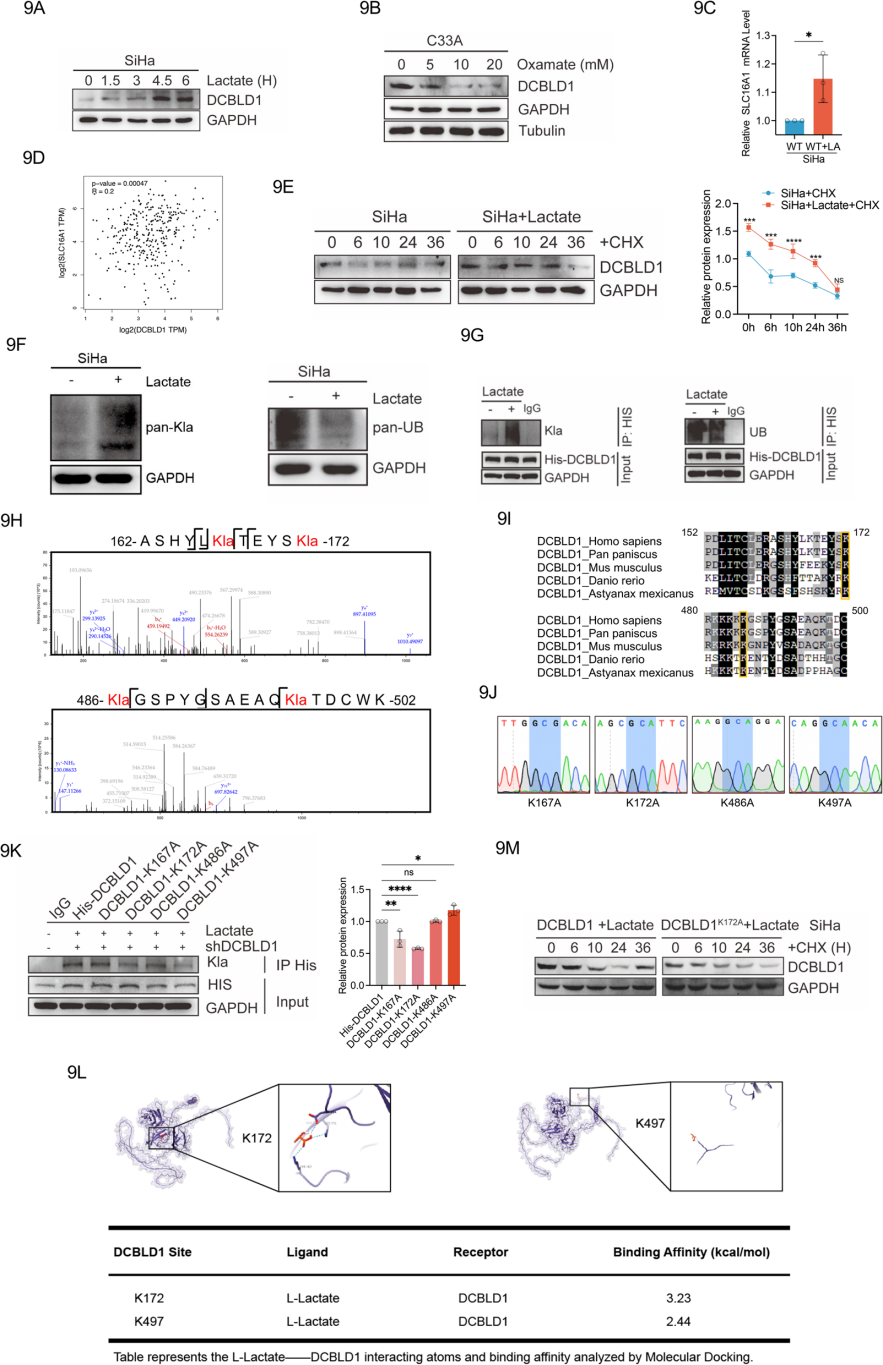
Lactylation stabilizes DCBLD1 expression. **A** Western blot analysis of the changes induced by sodium L-lactate (25 mM) on DCBLD1 at different times. **B** Western blot analysis of the changes induced by Oxamate (25 mM) on DCBLD1 at different times. **C** qPCR was performed to determine MCT1 (*SLC16A1*) mRNA levels in SiHa cells treated with sodium L-lactate (25 mM). **D** Analysis of the correlation between DCBLD1 and MCT1 (*SLC16A1*) using TCGA database. **E** Western blot analysis of DCBLD1 in SiHa cells treated with or without L-lactate (25 mM) and subsequently exposed to cyclohexane (CHX, 10 μg/ml) for the specified duration. The graph on the right is a statistical plot of the western blot. **F** Western blot analysis of pan-lactylation and pan-ubiquitination in SiHa cells, with and without sodium L-lactate. **G** Co-IP assay in SiHa cells transfected with the His-DCBLD1 plasmid, with or without L-lactate treatment for 6 h. **H** LC–MS/MS was performed to confirm the Kla of the DCBLD1 protein and identify specific lactylated modification sites. Specific lactylated modification sites (K167, K172, K486, and K497) were shown. **I** Species (*Home sapiens*, *Pan paniscus*, *Mus musculus*, *Danio rerio*, and *Astyanax mexicanus*) conservation analysis of potential lactylation modification sequence sites for DCBLD1. **J** Overlap-PCR was used for the construction of mutation plasmids. Sanger sequencing was used to verify whether lysine was mutated to alanine (GCG or GCA). (K) The mutant plasmid was re-expressed in SiHa cells knocked down for DCBLD1, and the DCBLD1 lactylation level was detected using immunoblotting. The graph on the right is a statistical plot of the western blot. (L) The 3D structure of DCBLD1 was predicted using the AlphaFold tool. Auto-Dock 4.0 was used to predict DCBLD1 binding to L-lactate. (M) Western blot analysis of DCBLD1 in SiHa-DCBLD1 KD re-expressing DCBLD1-WT or DCBLD1 K172A cell treated with L-lactate (25 mM) and subsequently exposed to cyclohexane (CHX, 10 μg/ml) for the specified duration. Data are presented as mean ± SD. Statistical significance was assessed using an unpaired t test (**C**), a two-way ANOVA with Šídák's multiple comparisons test (**E**), and a one-way ANOVA with Dunnett's multiple comparisons test (**K**). (****, *p* < 0.0001; ***, *p* < 0.001; **, *p* < 0.01; *, *p* < 0.05; ns, no significance)

Previous studies have reported that L-actate acts as a signaling molecule involved in protein expression [29]. L-lactate upregulated DCBLD1 expressions time-dependently (Fig. 9A). When a lactate dehydrogenase inhibitor (oxamate) was used, DCBLD1 expression was reduced (Fig. 9B). Monocarboxylate transporter 1 (MCT1; encoded by *Slc16a1*) is responsible for L-lactate transport. A significant increase in the expression of *Slc16a1 *was observed in SiHa cells after coculturing with L-lactate (Fig. 9C). Furthermore, *Slc16a1* expression exhibited a positive correlation with DCBLD1 expression based on information from TCGA database (Fig. 9D). We further examined the effect of L-lactate on the half-life of DCBLD1. L-lactate was used for 36 h, followed by CHX to inhibit cellular protein generation. The results showed that L-lactate prolonged the half-life of DCBLD1 compared to that in control-treated cells (Fig. 9E), indicating the involvement of L-lactate in regulating DCBLD1 protein stability.

Lactylation modification is recognized as a novel post-translational medication that is driven by L-lactate and lysine-dependent [14]. Previous studies have revealed that L-lactate directly promotes NUSAP1 lactylation to stabilize its expression [30]. Therefore, we explored the global expression of lactylation and ubiquitination. We found that L-lactate increased global lactylation while decreasing global ubiquitination concurrently (Fig. 9F). Subsequent IP results showed that L-lactate supplementation led to a significant increase in DCBLD1 immune complexes as detected using pan-lactylation antibodies. Simultaneously, it correspondingly reduced DCBLD1 immune complexes detected with pan-ubiquitination antibodies (Fig. 9G).

To validate the presence of Kla in the DCBLD1 protein, LC–MS/MS was employed to verify specific modification sites within the DCBLD1 protein. The findings revealed four potential lactylation sites (K167, K172, K486, and K497) (Fig. 9H). The G6PD protein is highly conserved. Therefore, we speculate that key regulatory sites susceptible to lactylation might also be conserved. Sequence alignments across diverse species revealed that two lysine residues (K172 and K486) were conserved (Fig. 9I). To identify the specific residues targeted for lactylation, we mutated each of the four putative lysine residues to alanine (A) (Fig. 9 J) and subsequently assessed the levels of lactylation. We found that the ectopic expression of K172A significantly inhibited lactylation (Fig. 9K). Molecular docking was also used to simulate the binding conformations of DCBLD1 and L-Lactate. Our analysis revealed that L-lactate binds to DCBLD at the K172 site with a high binding energy of 3.23 kal/mol, compared to a binding energy of 2.44 kal/mol at the K497 site (Fig. 9L). These findings suggest that K172 serves as the major lactylation modification site in DCBLD1. We next found that expression of DCBLD1 K172A shortened the half-life of DCBLD1 (Fig. 9M). Overall, our data indicates that L-lactate-mediated lactylation of DCBLD1 plays a direct stabilizing role in DCBLD1 expression.

## Discussion

Cervical cancer cells reside in an extremely nutrient-deprived and hypoxic tumor environment due to their rapid proliferation, hypovascularization, and desmoplastic reactions [31]. Thus, metabolic reprogramming is of utmost significance in meeting the energy and biosynthesis demands of cervical cancer cells, enabling them to survive in harsh environmental conditions [20]. In this study, metabolomic analysis revealed that DCBLD1 KD altered multiple metabolic pathways, including carbon, central carbon, and nucleotide metabolism. We identified G6PD-mediated oxiPPP as a crucial proliferative-related target of DCBLD1, establishing a link between DCBLD1 and the altered oxiPPP in cervical cancer. We then explored the underlying mechanism for the elevated DCBLD1 expression in two aspects. L-lactate serves as a signaling molecule, activating HIF-1α to promote DCBLD1 transcriptional expression. In contrast, L-lactate also directly increased DCBLD1 lactylation, thereby enhancing DCBLD1 post-transcriptional expression.

Metabolic abnormalities are the hallmarks of cancer. Most cancer cells augment glucose uptake for energy supply via glycolysis, even when oxygen is sufficiently available, a phenomenon known as the “Warburg effect” [32]. These metabolic alterations meet the demand for energy and nutrients essential for rapid genome replication [33]. HIF-1α is a key transcription factor promoting Warburg-like metabolism. HIF-1α regulates the expression of various enzymes within metabolic pathways [34], including pyruvate kinase M [35] and lactate dehydrogenase A [30]. In our study, HIF-1α was enriched in the DCBLD1 promoter, consequently increasing DCBLD1 expression. DCBLD1 overexpression upregulated G6PD-mediated oxiPPP.

Autophagy impairment contributes to tumor progression and survival by regulating metabolic enzyme expression. In hepatocellular carcinoma, hexokinase 2 (HK2), a rate-limiting enzyme in glycolysis, is recognized by the autophagy receptor SQSTM1 and subsequently subjected to selective degradation via the autophagy process. Therefore, impaired autophagy may be implicated in hepatocellular carcinoma development [26]. These findings underscore the role autophagy plays in maintaining cancer stem cell phenotypes by regulating the expression of enzymes involved in glycolysis. Fructose 2,6-biphosphatase 3 (Pfkfb3)^High^ Autophagy^Low^ phenotype was observed in metastatic breast cancer cells, whereas dormant breast cancer cells exhibited the Pfkfb3^Low^Autophagy^High^ phenotype [36]. These findings suggest that autophagy plays a crucial role in regulating the metabolic flux by modifying metabolic enzyme expression. In this study, autophagy impairment was consistently observed in cells overexpressing DCBLD1. Consequently, the pharmacological activation of autophagy led to a substantial reduction in G6PD expression in DCBLD1-overexpressing cells, thus inhibiting their growth, both in vitro and in vivo.

Lactylation, a novel post-translational modification, is facilitated by lactate [14]. Lactate, produced during glycolysis or transported into cells through MCT1, serves as a substrate for lactylation. While previous studies have primarily focused on histone protein lactylation, its influence extends to pro-fibrotic gene expression [37], macrophage polarization [38], somatic cell reprogramming [15] and nervous system modulation [39].

In a recent proteome-wide study, lactylation sites in numerous non-histone proteins were identified using affinity-directed mass spectrometry [40, 41]. In regulating macrophage function, Pyruvate kinase M2 lactylation promotes the transition from pro-inflammatory macrophages to a reparative phenotype [42]. Similarly, L-lactate-induced lactylation of high-mobility group box-1 (HMGB1) facilitates the release of HMGB1 by macrophage via exosome secretion during polymicrobial sepsis [43]. In oncological studies, nucleolar- and spindle-associated protein 1-mediated lactylation, in conjunction with lactate dehydrogenase A, forms a positive feedback loop that promotes pancreatic ductal adenocarcinoma metastasis [30]. In this study, we identified the DCBLD1 lactylation site and clarified its role in stabilizing DCBLD1 expression.

In summary, this study revealed the role of DCBLD1 in promoting the malignant progression of cervical cancer by activating G6PD-mediated PPP in vivo and in vitro. We also clarified the underlying mechanism for the elevated expression of DCBLD1 in cervical cancer at the transcriptional and post-transcriptional levels. L-lactate functions as a signaling molecule, activating HIF-1α to promote DCBLD1 transcription. Additionally, L-lactate inhibits DCBLD1 ubiquitination by directly enhancing DCBLD1 lactylation, particularly at lysine-172. We believe that inhibiting G6PD enzymatic activity through 6-An may prove pivotal in mitigating the oncogenic effects of DCBLD1.

### Supplementary Information

**Additional file 1: Supplementary Fig. 1.** DCBLD1 is highly expressed in cervical cancer. (A) Kaplan–Meier analysis of TCGA-MESO data showing overall survival of cervical cancer patients based on dichotomized expression of DCBLD1 (high or low relative to its median expression). (*n* = 273) Expression profiles and prognostic data of the DCBLD1 gene in 273 cervical cancer samples were obtained from the TCGA database. We then determined the median expression of DCBLD1. The samples were categorized into a high expression group (131) and a low expression group (142) based on whether the expression level of DCBLD1 was above or below the median. Finally, the expression data of the DCBLD1 gene in the 273 samples were merged with the clinical data, and survival curves were plotted (B) DCBLD1 expression in cancer and its corresponding normal samples was analyzed using RNA-seq data from the GEO database (GSE39001 and GSE75132). (C) DCBLD1 mRNA levels were assessed using qPCR in human normal cervical epithelial cells (HcerEpic) and cervical cancer cell lines, including HeLa, C33A, and SiHa. (D) DCBLD1 and G6PD correlation analysis using TCGA database (left). Analysis of the correlation between DCBLD1 and MCT1 (SLC16A1) using TCGA database (right). Data are presented as mean ± SD. Statistical significance was assessed using an unpaired t test (B) and a one-way ANOVA with Dunnett's multiple comparisons test (C). (***, *p* < 0.001; *, *p* < 0.05). **Supplementary Fig. 2.** Stable knockdown of DCBLD1 was established in both HeLa and C33A cells. (A) Western blot analysis of DCBLD1 in HeLa and C33A cells transfected with DCBLD1 shRNA or vector. GAPDH was used as a loading control. (B) DCBLD1 mRNA level in HeLa and C33A cells were determined using qPCR. (C) Scratch assay detected the cell migration. Data are presented as mean ± SD. Statistical significance was assessed using a one-way ANOVA with Dunnett's multiple comparisons test (B) or an unpaired t test (C). (****, *p* < 0.0001; ***, *p* < 0.001; *, *p* < 0.05). Scale bar: 200 µm.

**Additional file 2.**

## Data Availability

The datasets used and/or analyzed during the current study are available from the corresponding author on reasonable request.

## Abbreviations

oxiPPP: Oxidative PPP
non-oxiPPP: Non-oxidized PPP
DCBLD1: Discoidin, CUB, and LCCL domain-containing type I
G6PD: Glucose-6-phosphate dehydrogenase
BSA: Bovine Serum Albumin
CRKL: CRK-Like
GEO: Gene Expression Omnibus
LC–MS/MS: Liquid Chromatography-Mass Spectrometry
PCNA: Proliferating Cell Nuclear Antigen
PPP: Pentose Phosphate Pathway
ROS: Reactive Oxygen Species
S.D.: Standard Deviation
SDS: Sodium Dodecyl Sulfate
TCGA: The Cancer Genome Atlas
TOFMS: Time of Flight-Mass Spectrometry
WCE: Whole-Cell Extracts

## References

[1] UniProt Consortium T (2018). UniProt: the universal protein knowledgebase. Nucleic Acids Res.

[2] Nielsen H (2017). Predicting secretory proteins with signalP. Methods Mol Biol.

[3] Schmoker AM, Ebert AM, Ballif BA (2019). The DCBLD receptor family: emerging signaling roles in development, homeostasis and disease. Biochem J.

[4] Schmoker AM, Weinert JL, Markwood JM, Albretsen KS, Lunde ML, Weir ME (2020). FYN and ABL Regulate the Interaction Networks of the DCBLD Receptor Family. Mol Cell Proteomics.

[5] Aten TM, Redmond MM, Weaver SO, Love CC, Joy RM, Lapp AS (2013). Tyrosine phosphorylation of the orphan receptor ESDN/DCBLD2 serves as a scaffold for the signaling adaptor CrkL. FEBS Lett.

[6] Schmoker AM, Weinert JL, Kellett KJ, Johnson HE, Joy RM, Weir ME (2017). Dynamic multi-site phosphorylation by Fyn and Abl drives the interaction between CRKL and the novel scaffolding receptors DCBLD1 and DCBLD2. Biochem J.

[7] Fu L-L, Yan M, Ma M-X, Luo Y, Shao M, Gosau M (2022). DCBLD1 overexpression is associated with a poor prognosis in head and neck squamous cell carcinoma. Front Immunol.

[8] Cardin GB, Bernard M, Rodier F, Christopoulos A (2021). DCBLD1 is associated with the integrin signaling pathway and has prognostic value in non-small cell lung and invasive breast carcinoma. Sci Rep.

[9] Xu D, Wang Y, Liu X, Zhou K, Wu J, Chen J (2021). Development and clinical validation of a novel 9-gene prognostic model based on multi-omics in pancreatic adenocarcinoma. Pharmacol Res.

[10] Okada R, Goto Y, Yamada Y, Kato M, Asai S, Moriya S (2020). Regulation of Oncogenic Targets by the Tumor-Suppressive miR-139 Duplex (miR-139–5p and miR-139–3p) in Renal Cell Carcinoma. Biomedicines.

[11] Hanahan D (2022). Hallmarks of cancer: new dimensions. Cancer Discov.

[12] Liu B, Meng Q, Gao X, Sun H, Xu Z, Wang Y, Zhou H (2023). Lipid and glucose metabolism in senescence. Front Nutr.

[13] Li X, Yang Y, Zhang B, Lin X, Fu X, An Y (2022). Lactate metabolism in human health and disease. Signal Transduct Target Ther.

[14] Zhang D, Tang Z, Huang H, Zhou G, Cui C, Weng Y (2019). Metabolic regulation of gene expression by histone lactylation. Nature.

[15] Li L, Chen K, Wang T, Wu Y, Xing G, Chen M (2020). Glis1 facilitates induction of pluripotency via an epigenome-metabolome-epigenome signalling cascade. Nat Metab.

[16] Yang Z, Yan C, Ma J, Peng P, Ren X, Cai S (2023). Lactylome analysis suggests lactylation-dependent mechanisms of metabolic adaptation in hepatocellular carcinoma. Nat Metab.

[17] Chu YD, Cheng LC, Lim SN, Lai MW, Yeh CT, Lin WR (2023). Aldolase B-driven lactagenesis and CEACAM6 activation promote cell renewal and chemoresistance in colorectal cancer through the Warburg effect. Cell Death Dis.

[18] Meng Q, Zhang Y, Hao S, Sun H, Liu B, Zhou H (2022). Recent findings in the regulation of G6PD and its role in diseases. Front Pharmacol.

[19] TeSlaa T, Ralser M, Fan J, Rabinowitz JD (2023). The pentose phosphate pathway in health and disease. Nat Metab.

[20] Zeng Q, Zhao R-X, Chen J, Li Y, Li X-D, Liu X-L (2016). O-linked GlcNAcylation elevated by HPV E6 mediates viral oncogenesis. Proc Natl Acad Sci USA.

[21] Zeng Q, Chen J, Li Y, Werle KD, Zhao RX, Quan CS (2017). LKB1 inhibits HPV-associated cancer progression by targeting cellular metabolism. Oncogene.

[22] Hao S, Meng Q, Sun H, Yang X, Liu B, Zhang Y, et al. Human papillomavirus type 16 E6 promotes cervical cancer proliferation by upregulating transketolase enzymatic activity through the activation of protein kinase B. Mol Carcinog. 2023. 10.1002/mc.23656. 10.1002/mc.23656 37988232

[23] Chen J-L, Wu X, Yin D, Jia X-H, Chen X, Gu Z-Y, Zhu X-M (2023). Autophagy inhibitors for cancer therapy: small molecules and nanomedicines. Pharmacol Ther.

[24] Xie ZP, Klionsky DJ (2007). Autophagosome formation: Core machinery and adaptations. Nat Cell Biol.

[25] Tao T, Zhang P, Zeng Z, Wang M (2023). Advances in autophagy modulation of natural products in cervical cancer. J Ethnopharmacol.

[26] Jiao L, Zhang HL, Li DD, Yang KL, Tang J, Li X (2018). Regulation of glycolytic metabolism by autophagy in liver cancer involves selective autophagic degradation of HK2 (hexokinase 2). Autophagy.

[27] Deng H, Chen Y, Wang L, Zhang Y, Hang Q, Li P (2023). PI3K/mTOR inhibitors promote G6PD autophagic degradation and exacerbate oxidative stress damage to radiosensitize small cell lung cancer. Cell Death Dis.

[28] Xian H, Yang S, Jin S, Zhang Y, Cui J (2020). LRRC59 modulates type I interferon signaling by restraining the SQSTM1/p62-mediated autophagic degradation of pattern recognition receptor DDX58/RIG-I. Autophagy.

[29] Zhao Y, Li M, Yao X, Fei Y, Lin Z, Li Z (2020). HCAR1/MCT1 Regulates Tumor Ferroptosis through the Lactate-Mediated AMPK-SCD1 Activity and Its Therapeutic Implications. Cell Rep.

[30] Chen M, Cen K, Song Y, Zhang X, Liou Y-C, Liu P (2023). NUSAP1-LDHA-Glycolysis-Lactate feedforward loop promotes Warburg effect and metastasis in pancreatic ductal adenocarcinoma. Cancer Lett.

[31] Li B, Sui L (2021). Metabolic reprogramming in cervical cancer and metabolomics perspectives. Nutr Metab.

[32] Warburg O (1956). On the origin of cancer cells. Science (New York, NY).

[33] Chen Z, Han F, Du Y, Shi H, Zhou W (2023). Hypoxic microenvironment in cancer: molecular mechanisms and therapeutic interventions. Signal Transduct Target Ther.

[34] Kierans SJ, Fagundes RR, Malkov MI, Sparkes R, Dillon ET, Smolenski A (2023). Hypoxia induces a glycolytic complex in intestinal epithelial cells independent of HIF-1-driven glycolytic gene expression. Proc Natl Acad Sci USA.

[35] Zahid H, Subbaramaiah K, Iyengar NM, Zhou XK, Chen IC, Bhardwaj P (2018). Leptin regulation of the p53-HIF1α/PKM2-aromatase axis in breast adipose stromal cells: a novel mechanism for the obesity-breast cancer link. Int J Obes (Lond).

[36] La Belle FA, Calhoun BC, Sharma A, Chang JC, Almasan A, Schiemann WP (2019). Autophagy inhibition elicits emergence from metastatic dormancy by inducing and stabilizing Pfkfb3 expression. Nat Commun.

[37] Xiong J, He J, Zhu J, Pan J, Liao W, Ye H, et al. Lactylation-driven METTL3-mediated RNA m6A modification promotes immunosuppression of tumor-infiltrating myeloid cells. Mol Cell. 2022;82(9). 10.1016/j.molcel.2022.02.033 35320754

[38] Fang X, Zhao P, Gao S, Liu D, Zhang S, Shan M (2023). Lactate induces tumor-associated macrophage polarization independent of mitochondrial pyruvate carrier-mediated metabolism. Int J Biol Macromol.

[39] Hagihara H, Shoji H, Otabi H, Toyoda A, Katoh K, Namihira M, Miyakawa T (2021). Protein lactylation induced by neural excitation. Cell Rep.

[40] Wan N, Wang N, Yu S, Zhang H, Tang S, Wang D (2022). Cyclic immonium ion of lactyllysine reveals widespread lactylation in the human proteome. Nat Methods.

[41] Yang D, Yin J, Shan L, Yi X, Zhang W, Ding Y (2022). Identification of lysine-lactylated substrates in gastric cancer cells. iScience.

[42] Wang J, Yang P, Yu T, Gao M, Liu D, Zhang J (2022). Lactylation of PKM2 Suppresses inflammatory metabolic adaptation in pro-inflammatory macrophages. Int J Biol Sci.

[43] Yang K, Fan M, Wang X, Xu J, Wang Y, Tu F (2022). Lactate promotes macrophage HMGB1 lactylation, acetylation, and exosomal release in polymicrobial sepsis. Cell Death Differ.

