# Supplementary figures and images for: Lactylation stabilizes DCBLD1 activating the pentose phosphate pathway to promote cervical cancer progression
Source: J Exp Clin Cancer Res. 2024 Jan 31;43:36. doi: 10.1186/s13046-024-02943-x (PMC10829273; doi:10.1186/s13046-024-02943-x)

# Supplementary Figure 1

S1A

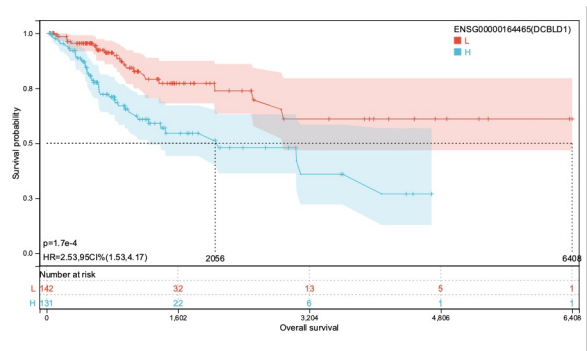

S1B

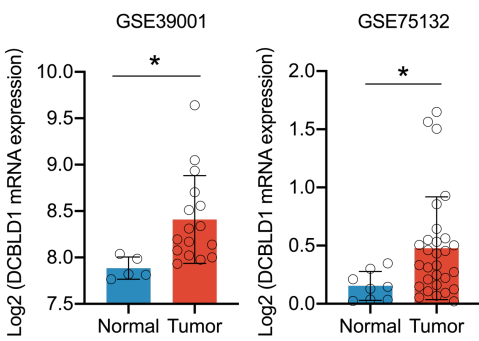

S1C

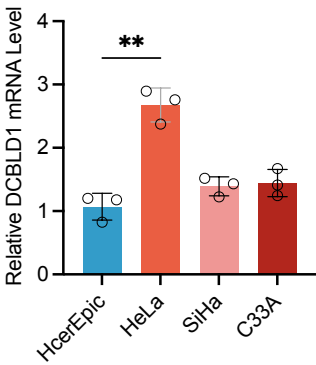

S1D

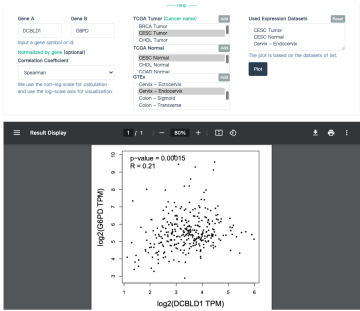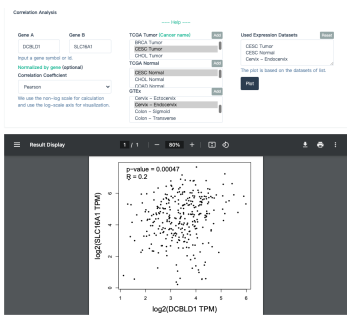

Supplementary Figure 2

S2A

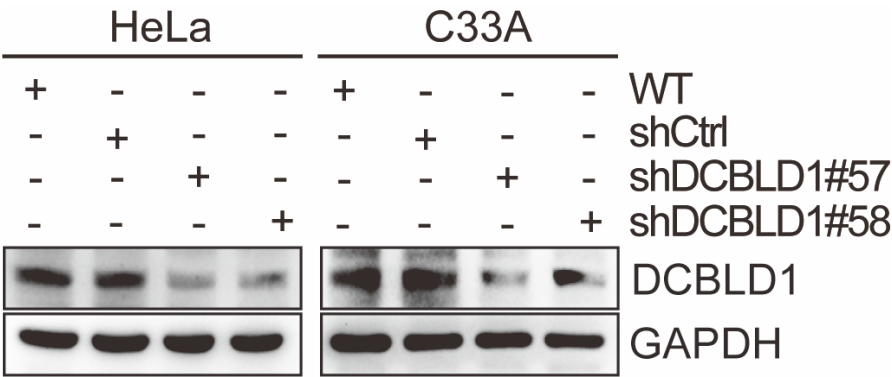

S2B

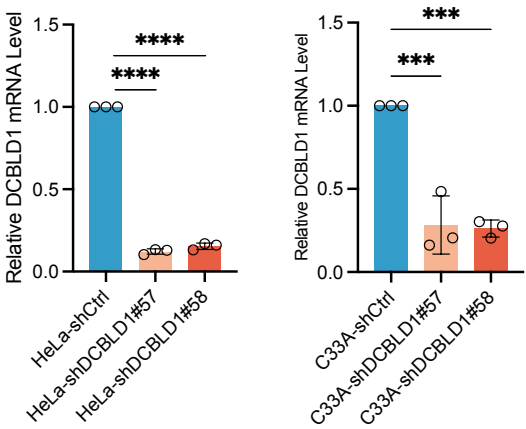

S2C

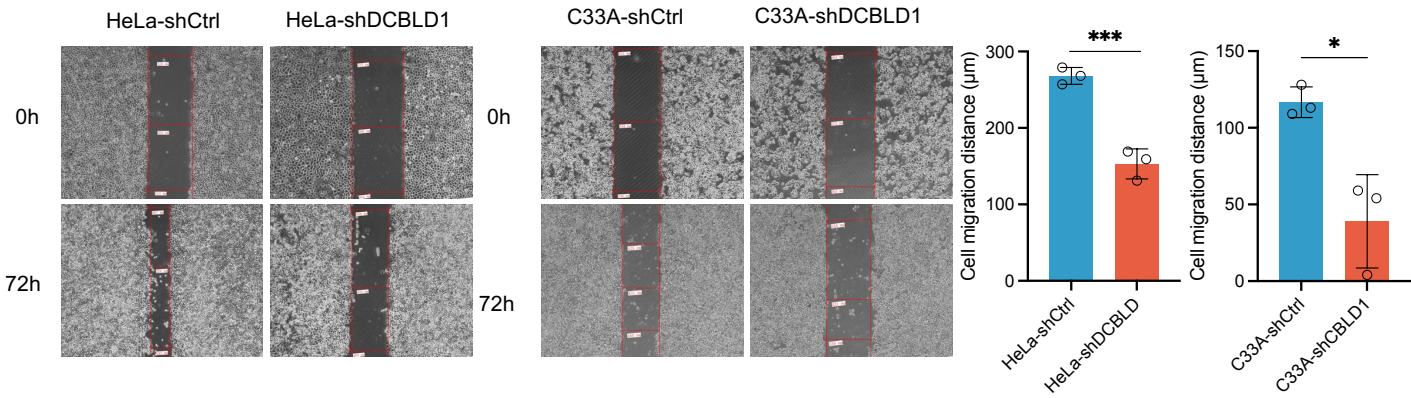

Supplement: Supplementary file 1 — Additional file 1: Supplementary Fig. 1. DCBLD1 is highly expressed in cervical cancer. (A) Kaplan–Meier analysis of TCGA-MESO data showing overall survival of cervical cancer patients based on dichotomized expression of DCBLD1 (high or low relative to its median expression). (n = 273) Expression profiles and prognostic data of the DCBLD1 gene in 273 cervical cancer samples were obtained from the TCGA database. We then determined the median expression of DCBLD1. The samples were categorized into a high expression group (131) and a low expression group (142) based on whether the expression level of DCBLD1 was above or below the median. Finally, the expression data of the DCBLD1 gene in the 273 samples were merged with the clinical data, and survival curves were plotted (B) DCBLD1 expression in cancer and its corresponding normal samples was analyzed using RNA-seq data from the GEO database (GSE39001 and GSE75132). (C) DCBLD1 mRNA levels were assessed using qPCR in human normal cervical epithelial cells (HcerEpic) and cervical cancer cell lines, including HeLa, C33A, and SiHa. (D) DCBLD1 and G6PD correlation analysis using TCGA database (left). Analysis of the correlation between DCBLD1 and MCT1 (SLC16A1) using TCGA database (right). Data are presented as mean ± SD. Statistical significance was assessed using an unpaired t test (B) and a one-way ANOVA with Dunnett's multiple comparisons test (C). (***, p < 0.001; *, p < 0.05). Supplementary Fig. 2. Stable knockdown of DCBLD1 was established in both HeLa and C33A cells. (A) Western blot analysis of DCBLD1 in HeLa and C33A cells transfected with DCBLD1 shRNA or vector. GAPDH was used as a loading control. (B) DCBLD1 mRNA level in HeLa and C33A cells were determined using qPCR. (C) Scratch assay detected the cell migration. Data are presented as mean ± SD. Statistical significance was assessed using a one-way ANOVA with Dunnett's multiple comparisons test (B) or an unpaired t test (C). (****, p < 0.0001; ***, p < 0.001; [file 13046_2024_2943_MOESM1_ESM.pdf]
