# Supplementary material for: Lactylation stabilizes DCBLD1 activating the pentose phosphate pathway to promote cervical cancer progression
Source: J Exp Clin Cancer Res. 2024 Jan 31;43:36. doi: 10.1186/s13046-024-02943-x (PMC10829273; doi:10.1186/s13046-024-02943-x)
Supplement: Supplementary file 2 — Additional file 2. [file 13046_2024_2943_MOESM2_ESM.docx]

**Materials and Methods**

**Plasmids**. His-DCBLD1, shDCBLD1, shRNA-G6PD, His-DCBLD1, LC3-GFP-mcherry and shHIF-1α constructs were purchased from GeneChem.

**Lentivirus production and stable knockdown cell lines construction.** To construct cell lines with stable knockdown endogenous DCBLD1 HIF-1α, and G6PD, lentivirus carrying shRNA were generated. Briefly, to produce lentivirus, lentiviral vector harboring shRNA, psPAX2 packaging plasmid, and pMD2.G envelope plasmid (Addgene) were co-transfected into HEK293T cells using Transfection Reagent (GeneCopoeia) according to the manufacturer’s instructions. Fresh medium was changed 24 h later and lentivirus-containing supernatant medium was collected 48 h after transfection. To construct stable knockdown cells, target cells were infected with harvested lentivirus-containing supernatant for 24 h and selected with 2mg/mL puromycin. Knockdown efficiency was confirmed by western blot.

**Cell culture, gene transfection, reagents, and** **materials.** HeLa, 293T, C33A, and SiHa cells were cultured in DMEM (Dalian Meilun Biotechnology Co., Ltd) supplemented with 10% (v/v) FBS (Dalian Meilun Biotechnology Co., Ltd) at 37 °C in 5% CO_2_. Rapamycin (AY-22989), Chloroquine (HY-17589A) and Cycloheximide (HY-12320) were purchased from MCE. MG-132 (C3348, APExBIO, Houston, USA) was purchased from APExBIO. Sodium L-lactate (Merck, 71718). Cell culture dishes/plates, 20-mmglass-bottom dishes, and transwell chambers were obtained from NEST Biotechnology Co. Ltd (Wuxi, China).

**Animal experiments**. The Animal Research Ethics Committee of Jilin University approved all animal experiments. Female BALB/c nude mice were purchased from Beijing HFK Bio-Technology Company (Beijing, China) and randomly assigned to experimental groups. For xenograft experiments, C33A or SiHa cell lines were transduced with the designated viruses expressing the proteins or shRNAs of interest. Equal numbers of stable cells were subcutaneously inoculated into nude mice. Starting on day 10 or 12 after injection, tumor volumes were measured every 2 or 3 days using a calliper and calculated using the following equation: volume = width × depth × length × 0.5.

**Western blot.** Cell lysates were prepared in RIPA buffer containing protease inhibitor (Solarbio). Equal amounts of protein were added into 10% gels by sodium dodecyl sulfate–polyacrylamide gel electrophoresis and transferred onto the polyvinylidene fluoride (PVDF) membrane. After blocking in a mixture of 5% bovine serum albumin (BSA) in Tris- buffered saline and Tween-20 (TBST) buffer, the mem- branes were incubated with specific primary antibodies at 4 °C overnight followed by secondary antibodies at room temperature for 2 h. Protein expression levels were visualized by enhanced chemiluminescence (ECL) detection system (Tanon). The following antibodies were used: p62 (abcam ab109012), DCBLD1 (abcam ab185216), mTOR (cst #2983), p-mTOR (cst #5536), LC3B (cst #83506), HIF-1α (cst #36169S), Pan-L-lactyl-lysine (PTM BioLabs PTM-1401RM), 6PGD (proteintech 14718-1-AP), Ubiquitin (cst #3936) 6*His (proteintech 66005-1-Ig), GAPDH (proteintech 60004-1-Ig), and G6PD (abcam ab210702).

**Immunoprecipitation.** Immunoprecipitation was performed as described previously (20). Briefly, about 500 µg of total cellular proteins were incubated with His antibody overnight at 4 °C followed by adding 30 µL of Protein A/G Magnetic Beads (MCE HY-K0202). The precipitates were washed seven times with lysis buffer and boiled in SDS sample buffer. The supernatant was subjected to immunoblotting with appropriate antibodies.

**Quantitation of mRNA expression via real-time qPCR**. Cells were washed with PBS and resuspended in 1 mL of TRIzol (Invitrogen, USA) and then stored at −80 °C. Total RNA extraction was conducted using TRIzol reagent according to the manufacturer’s protocol. RNA concentrations were measured using a nanodrop spectrophotometer (Thermo Fisher Scientific). Next, 1 μg of total RNA was transcribed into cDNA by the Hifair® Ⅲ 1st Strand cDNA Synthesis SuperMix Kit (YEASEN, China). qRT-PCR was then performed with the Hieff® qPCR SYBR Green Master Mix (YEASEN, China) on the 7300 Real-Time PCR Detection System (Applied Biosystems). The PCR conditions were 40 cycles of denaturation at 95°C for 10 seconds and annealing at 60°C for 31 seconds. Relative gene expression was calculated using the 2-ΔΔCt method. The expression level was normalized to that of tubulin. Sequences of primers used for real-time PCR. Tubulin Forward: CCCTAGAACAACGGCCTGA G. Reverse: GTTACCTGCCCCAGACTGAC. G6PD Forward: AAGAACGTGAAGCTCCCTGA. Reverse: AATATAGGGGATGGGCTTGG. GAPDH Forward: TGAAGGTCGGAGTCAACGGATT. Reverse: CTTCTCCATGGTGGTGAAGAC. DCBLD1 Forward: ACACAAGTGAAGTAACCGTCCG Reverse: TGCTACGTCTCTACAACCAGC. HiF1α Forward: AGAGGTTGAGGGACGGAGAT. Reverse: GCACCAAGCAGGTCATAGGT. MCT1 Forward: GGAACAAGCAAACGAGGCAG. Reverse: GCCGGCTGTTACCCAACTAA.

**Cell viability assay**. Overall, 2,000 cells were seeded in each well of a 96-well plate and allowed to adhere overnight. Cell viability was assessed 6 days later using the CCK-8 assay. The plates were read at a wavelength of 450 nm using a Synergy H1 microplate reader (BioTek Instruments).

**EdU incorporation assay**. EdU incorporation assays were performed using a Cell-Light EdU Apollo 488 In Vitro Imaging Kit (Beyotime Company, Shanghai, China) according to the manufacturer’s instructions. Images were captured using an Olympus DP70 microscope (Olympus), and the number of EdU-positive cells was counted.

**Immunofluorescence.** Cells, previously cultured on coverslips, were seeded 1 d before immunofluorescence analysis, reaching a final confluence of 70–80%. The cells were fixed with 4% paraformaldehyde for 10 min, permeabilized using 0.1% Triton X-100 for 5 min, blocked with 5% bovine serum albumin, and subsequently incubated with the specified antibodies. This was followed by staining with Texas Red-conjugated anti-rabbit IgG and fluorescein isothiocyanate-conjugated anti-mouse IgG. The cells were mounted with a DAPI-containing medium (Helixgen), and images were captured using a microscope (Olympus).

**Metabolic measurements**. Intracellular NADPH was measured using cell lysates with an NADPH assay kit (Beyotime) according to the manufacturer’s instructions. The intracellular GSH was measured using cell lysates with a GSH assay kit (Beyotime) according to the manufacturer’s instructions and normalized to the protein concentration. G6PD enzyme activity was determined by using a Solarbio kit (BC0265) according to the manufacturer’s instruction.

**Intracellular ROS measurement.** Intracellular ROS levels were assessed by detecting dichlorodihydrofluorescein, the cleavage product of carboxy-H2DCFDA (Invitrogen), following the manufacturer’s guidelines. Briefly, 2 × 10^5^ cells were initially seeded in 6-well plates. After 24 h, the cells were rinsed with PBS and then incubated with 5 μM carboxy-H2DCFDA for 30 min at 37°C. The cells were harvested, resuspended in PBS, and subjected to FACS analysis (BD Biosciences, USA; excitation and emission at 490 and 530 nm, respectively).

**Transwell Cell Migration/Invasion Matrigel Assays**. C33A and HeLa cells alone or transduced with shR-DCBLD1, or shR-Ctrl were placed in the upper chamber of the Transwell Membrane Inserts or precoated Matrigel Chambers from BD Biosciences. After 48 h, migrated/invasive cells were stained with 1% crystal violet and quantified under a microscope.

**LC-MS analysis of cell metabolites.** Approximately 5 × 10^6^ cells were washed twice with cold PBS, and polar metabolites were immediately extracted with ice-cold 80% methanol. Samples were subjected to freeze–thaw cycles or sonication to extract the metabolites. The supernatants were collected and dried. The powder containing metabolites was dissolved in 80% methanol to run LC-MS. For the kinetic LC-MS analyses, a Shimadzu Nexera ×2 UHPLC combined with a Sciex 5600 Triple Time of Flight-Mass Spectrometry (TOFMS) was used, which was controlled by Sciex Analyst 1.7.1 instrument acquiring software. A Supelco Ascentis Express HILIC Acquity UPLC BEH Amide (150 cm × 2.1 mm, 1.7 μm) column was used with mobile phase (A) consisting of 5 mM ammonium formate and 0.05% formic acid; mobile phase (B) consisting of 90% acetonitrile (ACN) and 10% water. Gradient program: mobile phase (A) was held at 15% for 0 min and then increased to 30% in 7 min; then to 60% in 6 min and held for 1 min before returning initial condition. The column was held at 40 °C and 5 μl of sample was injected into the LC-MS with a flow rate of 0.2 ml/min. Automatic calibrations of TOFMS were achieved with average mass accuracy of < 2 ppm. Data Processing Software included Sciex PeakView 2.2, MasterView 1.1, and MultiQuant 3.0.2.

**Immunohistochemistry.** Immunohistochemical staining of cervical cancer tissues was performed at the Histomorphology Platform of Jilin University following the manufacturer’s standard protocol. Briefly, the tissues were fixed with 4% paraformaldehyde at room temperature. Following paraffin embedding, the tissue paraffin blocks were sectioned into 4 mm slices. The sections were deparaffinized with xylene and subsequently rehydrated through a series of decreasing ethanol concentrations. After conducting antigen retrieval through high-pressure heat treatment, the slides were treated with 3% H2O2 for 10 min. Subsequently, the tissue slides were blocked in 5% goat serum for 30 min and left to incubate with primary antibodies overnight at 4°C. The following day, the slides were washed thrice with PBS. HRP-conjugated secondary antibody was used and incubated for 30 min at 37°C. The slides were then washed thrice with PBS. Subsequently, the slides were subjected to color development using 3, 3’-diaminobenzidine (DAB). Images were captured using a microscope (OLYMPUS BX51). The staining index was based on the staining intensity, which was graded as “−,” no staining; “+,” weak staining; “++,” moderate staining; and “+++,” strong staining. Samples that scored as “−” or “+” were considered as low expression and those scored as “++” or “+++” as high expression. All stained slides were ob- served and scored by two pathologists. If the staining interpretation differed between the two investigators, the data for the slide were discarded. For determining the H score, stained tissues were scored by calculating the product of the intensity level and the percentage of cells staining at that level (0, negative; 1, weak; 2, moderate; 3, strong). An H score was then calculated by summing the individual intensity level scores.

**LC-MS for lactylation detection.** HEK293T cells were transfected with the His-DCBLD1 plasmid for 24 h and then exposed to L-lactate for 24 h. Cell lysates were treated with 1% sodium dodecyl sulfate (SDS) before immunoprecipitation using an antibody targeting the His epitope tag. SDS-PAGE was performed, followed by Coomassie blue staining and the excision of bands for detection. Mass spectrometry (LTQ Orbitrap Elite, Thermo Fisher Scientific) was used to identify the lactylation of the DCBLD1 protein.

**Chromatin immunoprecipitation and reporter assays.** For chromatin immunoprecipitation assays, cells were crosslinked with 1% formaldehyde for 15 min at room temperature. The crosslinking reaction was halted by adding 12 nM glycine (final concentration). Cell lysates were sonicated to generate DNA fragments with an average size of <1,000 bp and these fragments were subjected to immunoprecipitation with the indicated antibodies. The bound DNA fragments were eluted and then amplified through PCR. Primer pairs used were as follows: HIF-1α, 5’-ATCATCGGGTGGAGTCCTTC-3’ and 5’-GCACAGTGAACAGGTGGGT-3’’.

In the reporter assay, the DCBLD1 genomic fragments containing the wild-type or mutant HIF-1α- binding region were cloned into a pGL3-basic vector (Promega, catalog No: E1751). Luciferase reporter assays were performed as previously described. Briefly, reporter plasmids were transfected into DCBLD1-null 293T and SiHa cells along with a Renilla luciferase plasmid. After 24 h of transfection, the luciferase activity was assessed using a dual-luciferase assay system (Promega, catalogue number: E1910). Transfection efficiency was normalized to the Renilla luciferase activity.

**Molecular Docking**. Auto-Dock 4.0 was used to dock L-Lactate (PubChem CID: 5460161) into protein DCBLD1. 3D structure of DCBLD1 was predicted by AlphaFold tool. In this study, the binding of L-Lactate and DCBLD1 was simulated. Before the docking simulation, DCBLD1 was placed into the substrate binding site of L-Lactate as the start point of docking. The conformation with the lowest binding energy of DCBLD1 was considered as the L-Lactate-bound conformation. By comparing the binding energy and conformation of the L-Lactate with DCBLD1 K172/497, We inferred that DCBLD1 K172 is more likely to undergo lactylation modification compared to K497.

**Statistical Analysis**. Data were obtained from at least 3 biological individual experiments and presented as the mean ± standard deviation (SD). ImageJ was used to quantify the western blotting and immunofluorescence results. Statistical data were analyzed using GraphPad Prism 9.5. The Pearson’s χ2-test was used to analyze the distribution difference of DCBLD1 and G6PD staining among human cervical tissues and xenograft tumor model tissues. A two-tailed Student's t-test was used for the comparison between two groups. ANOVA was applied for the comparisons among three or more groups. P < 0.05 was considered to represent a significant difference. *p < 0.05, **p < 0.01, ***p < 0.001.
